# UGDH promotes tumor-initiating cells and a fibroinflammatory tumor microenvironment in ovarian cancer

**DOI:** 10.1186/s13046-023-02820-z

**Published:** 2023-10-19

**Authors:** Brittney S. Harrington, Rahul Kamdar, Franklin Ning, Soumya Korrapati, Michael W. Caminear, Lidia F. Hernandez, Donna Butcher, Elijah F. Edmondson, Nadia Traficante, Joy Hendley, Australian Ovarian Cancer Study, Madeline Gough, Rebecca Rogers, Rohan Lourie, Jyoti Shetty, Bao Tran, Fathi Elloumi, Abdalla Abdelmaksoud, Madhu Lal Nag, Krystyna Mazan-Mamczarz, Carrie D. House, John D. Hooper, Christina M. Annunziata

**Affiliations:** 1 Women’s Malignancies Branch, National Cancer Institute, National Institutes of Health, Bethesda, MD 20892 USA; 2 Molecular Histopathology Laboratory, Frederick National Laboratory for Cancer Research, NCI, Frederick, MD 21702 USA; 3 Peter MacCallum Cancer Centre, Melbourne, VIC Australia; 4 Sir Peter MacCallum Department of Oncology, The University of Melbourne, Melbourne, VIC Australia; 5 Mater Brisbane Hospital, Mater Health Services, South Brisbane, QLD 4101 Australia; 6 Mater Research Institute, The University of Queensland, Translational Research Institute, Woolloongabba, QLD 4102 Australia; 7 CCR Sequencing Facility, Leidos Biomedical Research, Inc., Frederick National Laboratory for Cancer Research, Frederick, MD 21701 USA; 8 Collaborative Bioinformatics Resource (CCBR), Center for Cancer Research (CCR), National Cancer Institute (NCI), National Institutes of Health, Bethesda, MD USA; 9 Advanced Biomedical Computational Science, Frederick National Laboratory for Cancer Research, Frederick, MD USA; 10 Functional Genomics Lab, National Center for Advancing Translational Sciences, National Institutes of Health, Bethesda, MD 20892 USA; 11 Present address: Department of Biology, San Diego State University, San Diego, CA 92182 USA

**Keywords:** UGDH, Ovarian cancer, Molecular subtypes, Mesenchymal, Tumor microenvironment

## Abstract

**Background:**

Epithelial ovarian cancer (EOC) is a global health burden, with the poorest five-year survival rate of the gynecological malignancies due to diagnosis at advanced stage and high recurrence rate. Recurrence in EOC is driven by the survival of chemoresistant, stem-like tumor-initiating cells (TICs) that are supported by a complex extracellular matrix and immunosuppressive microenvironment. To target TICs to prevent recurrence, we identified genes critical for TIC viability from a whole genome siRNA screen. A top hit was the cancer-associated, proteoglycan subunit synthesis enzyme UDP-glucose dehydrogenase (UGDH).

**Methods:**

Immunohistochemistry was used to characterize UGDH expression in histological and molecular subtypes of EOC. EOC cell lines were subtyped according to the molecular subtypes and the functional effects of modulating UGDH expression in vitro and in vivo in C1/Mesenchymal and C4/Differentiated subtype cell lines was examined.

**Results:**

High UGDH expression was observed in high-grade serous ovarian cancers and a distinctive survival prognostic for UGDH expression was revealed when serous cancers were stratified by molecular subtype. High UGDH was associated with a poor prognosis in the C1/Mesenchymal subtype and low UGDH was associated with poor prognosis in the C4/Differentiated subtype. Knockdown of UGDH in the C1/mesenchymal molecular subtype reduced spheroid formation and viability and reduced the CD133 + /ALDH ^high^ TIC population. Conversely, overexpression of UGDH in the C4/Differentiated subtype reduced the TIC population. In co-culture models, UGDH expression in spheroids affected the gene expression of mesothelial cells causing changes to matrix remodeling proteins, and fibroblast collagen production. Inflammatory cytokine expression of spheroids was altered by UGDH expression. The effect of UGDH knockdown or overexpression in the C1/ Mesenchymal and C4/Differentiated subtypes respectively was tested on mouse intrabursal xenografts and showed dynamic changes to the tumor stroma. Knockdown of UGDH improved survival and reduced tumor burden in C1/Mesenchymal compared to controls.

**Conclusions:**

These data show that modulation of UGDH expression in ovarian cancer reveals distinct roles for UGDH in the C1/Mesenchymal and C4/Differentiated molecular subtypes of EOC, influencing the tumor microenvironmental composition. UGDH is a strong potential therapeutic target in TICs, for the treatment of EOC, particularly in patients with the mesenchymal molecular subtype.

**Supplementary Information:**

The online version contains supplementary material available at 10.1186/s13046-023-02820-z.

## Background

Epithelial ovarian cancer (EOC) remains the most lethal gynecologic malignancy, with 19, 710 new cases and 13,270 deaths estimated in the United States in 2023 [1]. EOC is defined by a high level of heterogeneity, diagnosis at an advanced stage, and a high rate of disease relapse [2]. Survival rates of Stage 1 disease, when cancerous tissue is confined to the ovary, stay promising with percentages as high as 90% after five years [2]. However, disease metastasis, often to the omentum and peritoneum, complicates treatment and dramatically reduces survival 5-year survival rates to 30% [3, 4]. Furthermore, the stratification of high-grade ovarian cancers by molecular subtype reveals differences in survival, disease burden and surgical complexity. The mesenchymal molecular subtype of ovarian cancer has the worst overall survival and is associated with poorer surgical outcomes due to increased upper abdominal metastases, suboptimal debulking and severe postoperative complications [5–7].

The presence of malignant ascites allows dissemination of EOC tumor cells as spheroids to other peritoneal and abdominal sites [8]. EOC spheroids harbor stem-like tumor-initiating cells (TICs) and present significant challenges to successful therapy of metastatic EOC as they promote chemoresistance and disease recurrence [9–11]. Furthermore, the complex and immunosuppressive tumor microenvironment (TME) of EOC presents significant challenges to treatment and promotes survival and metastasis of TICs [12]. Extracellular matrix (ECM) proteoglycans abundant in the EOC microenvironment promote metastasis, bind to and moderate the activity of cytokines and chemokines, and modulate the interactions between heterotypic cell types [13].

We hypothesize that TICs, supported by this complex TME, are a target for therapeutic eradication. In this study, we identified genes essential for spheroid survival and investigated the enzyme UDP-glucose-6 dehydrogenase (UGDH). Functionally, UGDH promotes the synthesis of glycosaminoglycans and proteoglycans which helps maintain the integrity of the extracellular matrix [14, 15]. UGDH produces the substrates necessary for hyaluronic acid by oxidizing the nucleotide sugar UDP-glucose, to UDP-glucoronate [16] and is involved in drug and hormone metabolism through glucuronidation [17, 18]. UGDH has been associated with promoting cancers of the lung [19, 20], glioblastoma [21, 22], colon [23], prostate [24], breast [25–27] and ovary [28].

Here we examined the expression and localization of UGDH in tissue microarrays of EOC histotypes mucinous, endometrioid, clear cell and serous, as well as in the molecular subtypes of high-grade cancers [29] and report its prognostic value. We show UGDH promotes TIC survival and that targeting this enzyme in the highly aggressive mesenchymal molecular subtype reduces viability post-chemotherapy in vitro and tumor growth in vivo*.* Further, alteration of UGDH in spheroids influenced the gene expression of mesothelial cells in co-culture, remodeling the ECM and TME. UGDH is a strong potential therapeutic target in TICs for the treatment of metastatic and recurrent EOC, especially of the mesenchymal subtype.

## Materials and methods

### Antibodies and reagents

Carboplatin (Cat# 2626) was purchased from Tocris Bioscience (Minneapolis, MN) and dissolved in ultra-pure water. Propidium Iodide (R37169) was from Thermo Fisher Scientific (Waltham, MA) and AnnexinV-FITC (556,420) was from BD Biosciences (San Jose, CA). UGDH (HPA036656) was from Atlas Antibodies (Stockholm, Sweden), E-cadherin (4065) was from Cell Signaling Technology (Danvers, MA), and Vimentin (V6389) and GAPDH (MAB374) antibodies were from Millipore Sigma (Burlington, MA). Doxycycline (DOX) used for in vitro studies was from Millipore Sigma (D5207, Burlington, MA). Inducible shRNA for knockdown of human UGDH (SMARTvector Inducible Lentiviral shRNA) and human UGDH for over-expression (Precision LentiORF) were purchased from Horizon Discovery (Cambridge, United Kingdom).

### Tissue microarray immunohistochemistry and quantification

A TMA containing duplicate cores from archival samples of 96 HGS cases was generated as previously described [30]. IHC staining for UGDH was performed using Novolink Polymer Detection Systems kit (RE7150-CE, Leica Microsystems, Mt Waverley, Australia) according to the manufacturer’s instructions. Briefly, slides were deparaffinized in xylene followed by graded alcohols then blocking for endogenous peroxidases and non-specific proteins (5 min at room temperature). Antigen retrieval was performed using Citrate Buffer pH 6.0 (005000, Thermo Fisher Scientific) at 110 ºC for 15 min, followed by overnight incubation at 4 ºC with the primary antibody (UGDH, 1:750). The secondary antibody and detection steps were performed using the Novolink Polymer Detection Systems Kit. Staining was scored by a pathologist (R.L) for intensity of staining and percentage of tumor cells expressing UGDH, providing an overall score of negative (score 0), weak (score 1), moderate (score 2) or strong (score 3). Four TMAs containing duplicate cores from 1: clear cell ovarian cancer, 2: mucinous ovarian cancer, 3: endometrioid ovarian cancer, 4: molecular subtyped ovarian cancer (Australian Ovarian Cancer Study, http://www.aocstudy.org/) were evaluated for expression of UGDH. IHC staining was performed at the Molecular Histopathology Laboratory (NCI, Frederick MD) on Leica Biosystems’ BondRX autostainer with the following conditions: Epitope Retrieval 1 (Citrate buffer) 20 min, UGDH (1:750, 30 min), and the Bond Polymer Refine Detection Kit (with omission of the Post Primary Reagent), (DS9800 Leica Biosystems Deer Park, IL,). Rabbit polyclonal isotype control (ab37415, Abcam Waltham, MA) was used in place of UGDH for the negative control. Slides were removed from the autostainer, dehydrated through ethanols, cleared with xylenes, and coverslipped. Positive control tissue included ovarian, prostate, and breast carcinoma tissue. Negative controls were performed for each TMA evaluated; negative controls include replacing the anti-UGDH antibody with nonspecific antibody of the same isotype (isotype control) taken from the same host. Slides were digitized with an Aperio ScanScope XT (Leica Microsystems, Buffalo Grove, IL) at 400X in a single z-plane. Aperio whole-slide images were evaluated and a threshold for positivity was determined using known positive controls by a board-certified pathologist. Cell detection algorithms were run to assess the positive cells for two separate outputs: cytoplasmic or membranous positive and nuclear positivity. Machine learning, random forest algorithms were trained for each tissue array to classify each cell detection as either epithelial or stromal; UGDH staining was separately quantified based on epithelial (tumor) or stromal. Stromal staining of UGDH was not observed, therefore only the epithelial/tumor staining expression was quantified. The staining intensity was scored using a scale of 0–3: 0 for no staining, 1 for mild staining, 2 for moderate, and 3 for strong staining and tumor H-score [31] was calculated using QuPath [32] as follows: H-score = [1 × (% cells 1 +) + 2 × (% cells 2 +) + 3 × (% cells 3 +)].

### Histopathology, immunohistochemistry and analysis of mouse xenograft tissues

Formalin fixed paraffine embedded (FFPE) tissues were processed for hematoxylin and eosin staining following standard protocols at the Molecular Histopathology Laboratory, NCI-Frederick, MD, USA. Masson’s Trichrome staining was performed following standard protocols, briefly, sections were de-paraffinized and hydrated to distilled water, mordant in Bouins solution for 1 h at 56 ℃ then rinsed in distilled water. Weigert’s Hematoxylin was added for 10 min, then washed in water 10 min, followed by staining with Bierbrich Scarlet-Acid Fuchsin Solution for 6 min, rinsed with distilled water. Then stained with Phosphomolybdic–Phosphotungstic acid for 20 min, then Aniline Blue for 15 min, rinsed in distilled water, then 1% Glacial Acetic Acid for 5 min. Sections were dehydrated, cleared and mounted on slides. Whole slide imaging was performed at 200 × with a Leica Aperio Scanner. Annotations were made to include tumor tissue and exclude tumor necrosis or adjacent normal tissues. Tumor necrosis was estimated using image analysis tissue classifier and reported as a percentage of the total tissue area. Masson’s Trichrome staining was quantified using HALO image analysis platform (Indica Labs, Albuquerque, NM), in viable tissue using a positive pixel algorithm and reported as the percentage of trichrome positive pixels. Serial sections from FFPE tissues were stained for CD45, CD206, iNOS, and Iba-1 using chromogenic IHC. Staining for CD45 (550,539, BD Biosciences, San Jose, CA) was performed at a dilution of 1:100, overnight at room temperature after antigen retrieval in citrate buffer. Staining for CD206 (24,595, Cell Signaling Technology, Danvers, MA) was performed at a dilution of 1:400 for 1 h, after heat induced epitope retrieval (HIER) in EDTA. Staining for iNOS (Ab15323, Abcam, Cambridge, MA) was performed at a dilution of 1:50, after HIER in EDTA. Staining for Iba-1 (CP 290, Biocare, Pacheco, CA) was performed at a dilution of 1:500 after HIER in citrate buffer. For quantification of IHC, image analysis algorithms were utilized to quantify the number of positive cells per mm^2^ within the tumor, excluding necrosis. Thresholds for positive were determined using positive and negative controls. Two approaches were used to quantify staining and provide complimentary results: cell-detection based quantification providing the number of cells per mm^2^ and positive pixel quantification providing the number of positive pixels within each tumor.

### Cell lines and culture conditions

Ovarian cancer lines were obtained as gifts, or from ATCC or NCI-60 as described and were cultured as described [33]. TIC-enriching spheroid culture conditions are previously described [34–36]. Briefly, spheroids were generated by maintaining cells in ultra-low attachment (ULA) plates or flasks (Corning, Corning, NY) in defined medium. Experiments involving the TIC-enriched spheroid populations were grown for 3 days in defined medium in ULA plates before treatments were performed. LP3 mesothelial cells were obtained from the Coriell Institute and were grown in 1:1 Ham’s F12: Medium 199 containing 15% (v/v) FCS, penicillin (100 units per ml) and streptomycin (100 units per ml), 10 ng/ml EGF and 0.4 µg/ml hydrocortisone (Millipore Sigma, Burlington, MA). Human primary ovarian fibroblasts were purchased from Cell Biologics (H-6072, Chicago, IL) and were cultured according to the manufacturer’s instructions, on gelatin-coated (6950, Cell Biologics, Chicago, IL) tissue culture flasks in Fibroblast Medium (M2267, Cell Biologics, Chicago, IL). All cultures were maintained at 37 °C in 5% CO_2_.

### Whole genome siRNA screen

The whole genome RNAi screen was performed at the Functional Genomics Lab (Rockville, MD), previously known as the Trans-NIH RNAi Facility (TNRF) as previously described [37, 38]. Briefly, the RNAi screen targeting 10,415 druggable genes (three individual siRNAs per gene) was conducted using OV90 cells and the Silencer® Select Human Druggable Genome siRNA Library Version 4 (Ambion Thermo Fisher Scientific, Waltham, MA), in absence or presence of bardoxolone methyl. Adherent cells screening was carried out in 384-well white, solid, flat-bottom tissue culture plates (Corning, Corning, NY) while for spheroids screening 384-well black, clear, round-bottom ultra-low-attachment spheroid microplates were used (Corning, Corning, NY). Microplates were pre-stamped with one siRNA per well (2 µL, 400 nM) and, then 20ul of serum-free media containing Lipofectamine RNAiMax (Thermo Fisher Scientific, Waltham, MA) was added to each well. After 45 min incubation at room temperature, cells were added to wells in 20 µL media containing 20% FBS. Cells were cultured for 96 h, then cell viability was measured by the CellTiter-Glo Luminescent Cell Viability Assay (Promega, Madison, WI) with using EnVision Plate Reader (PerkinElmer, Boston, MA). Data analysis was performed as described [39]. To rank genes that inhibited spheroid viability, the *Z*‐score was calculated for each gene as: *Z* = (*x* − *μ*)/*σ*, *x* is the experimental value; *μ* is the median screen value; and *σ* is the standard deviation for the screen [40].

### RNA-sequencing alignment and analysis of ovarian cancer cell lines for molecular subtypes

Ovarian cancer cell lines were cultured in adherent conditions, and RNA was harvested according to the manufacturer’s instructions (74,104, Qiagen, Germantown, MD). Sequencing was performed at the CCR Sequencing Facility (Leidos Biomedical Research, Frederick, MD). RNA-seq libraries were generated using TruSeq RNA Stranded Total RNA Library Prep Kits (TruSeq Illumina RS-122–2201) and sequenced on a total of 10 Hiseq 2500 lanes using the 125 bp paired-end sequencing method (Illumina, San Diego, CA). Both reads of each sample were trimmed for adapters and low-quality bases using Trimmomatic software and aligned with reference human hg19 genome and ensemble v70 transcripts using Tophat software as stranded libraries. The sequencing quality of the reads was assessed per sample using FastQC (version 0.11.5) (http://www.bioinformatics.babraham.ac.uk/projects/ fastqc/), Preseq (version 2.0.3) [41], Picard tools (version 1.119) (https://broadinstitute.github.io/ picard/) and RSeQC (version 2.6.4) (http://rseqc.sourceforge.net/) [42]. Reads were then trimmed using Cutadapt (version 1.14) (https://cutadapt.readthedocs.io/en/stable/) [43] prior to mapping to the hg19 human genome using STAR (version 2.5.2b) (https://github.com/alexdobin/STAR) [44] in two-pass mode. Overall expression levels were quantified using RSEM (version 1.3.0) (https://deweylab.github.io/RSEM/) [45]. For normalization limma voom (version 3.48.3) [46] was used. For gene set enrichment, GSVA [47] was used using default parameters against 4 signatures from 4 subclusters [29] and used to create hierarchal clustering heatmap.

### Western blot analysis

Whole cell lysates were collected in lysis buffer: RIPA buffer (Thermo Scientific, Waltham, MA) containing 1 × protease and phosphatase inhibitor cocktail (78,440, Thermo Fisher Scientific, Waltham, MA). After a brief incubation on ice, the lysates were homogenized by passing the samples through 26-G needles, followed by centrifugation at 16,000 g, 4℃, for 20 min to collect the supernatant. Protein concentration was quantified by microbicinchoninic acid assay (23,227, Thermo Fisher Scientific, Waltham, MA). Lysates (30 µg) were separated by SDS-PAGE under reducing conditions, transferred to nitrocellulose membranes, and blocked in Intercept TBS blocking buffer (927–66,003, LI-COR Biosciences, Lincoln, NE). Membranes were incubated with primary antibodies diluted in blocking buffer overnight at 4 °C, (UGDH 1:1000), (GAPDH 1:10,000), washed with Tris-buffered saline containing 0.1% Tween 20 (TBST), then incubated with fluorescent secondary mouse or rabbit IgG antibodies (IRDye, LI-COR Biosciences, Lincoln, NE). Images were generated using the Odyssey system and software (LI-COR Biosciences, Lincoln, NE).

### Brightfield and Immunofluorescent microscopy

Brightfield images of adherently cultured and DOX-induced OV90, and ACI23 cells were taken on a Nikon Eclipse Ts2 inverted microscope with NIS elements software (Nikon, Melville, NY) at 10 × and 20 × magnification. For immunofluorescent staining, OV90 cells were plated on glass coverslips and cultured for 3 days in DOX-containing media. Cells were washed with phosphate-buffered saline (PBS), fixed with 4% paraformaldehyde for 20 min, permeabilized with 0.3% Triton X-100, blocked with 10% goat serum in PBS for 1 h at room temperature, and then incubated with primary antibodies (1:500) in 10% goat serum in PBS overnight in a humidified chamber, at 4 °C. The following day, coverslips were washed three times with PBS, incubated with fluorescent secondary antibodies (A-11034, A-21236, Thermo Scientific, Waltham, MA) at 1:2000, for 1 h at room temperature protected from light, washed three times with PBS and mounted onto glass slides with Fluoroshield with DAPI (F6057, Millipore Sigma, Burlington, MA). Images were taken on an inverted Nikon Ti2-E microscope (Nikon, Melville, NY), equipped with a Yokogawa SoRa CSU-W1 spinning disk unit (Yokogawa, Sugar Land, TX) and a BSI sCMOS camera (Teledyne Photometrics, Tuscon, AZ) at 60 × magnification. Signal intensity was measured using NIS Elements AR software (Nikon, Melville, NY).

### Cell viability

Cell viability was assessed as previously described [35, 36] using CellTiter-Glo (Promega, Madison, WI) according to manufacturer’s instructions.

### Sphere formation

Sphere formation was performed as previously described [35, 36]. OV90 and HEYA8 cells were seeded at 2000 cells/well in 96-well ULA plates (3474, Corning, NY), in TIC-enriching medium (TEM) with 1 µg/mL doxycycline (DOX) for 7 days, fresh culture medium containing growth factors was replenished every 48 h. ACI23 and SKOV3 cells were seeded at 1000 cells/well in 96-well ULA plates in TEM for 7 days, fresh culture medium containing growth factors was replenished every 48 h. After 7 days the spheroids were incubated with DRAQ5 (62,254, Thermo Fisher Scientific, Waltham, MA, USA) at 1 µM for 15 min prior to imaging as described [35]. Quantification of spheroids was performed using NIS Elements software (Nikon, Melville, NY), as described [35] and the number of spheroids measuring an area of > 1000 μm^2^ were counted.

### Colony formation

The colony formation assay was performed according to the manufacturer’s instructions (CBA-130, Cell Biolabs, San Diego, CA). Briefly, a base layer of agar was plated and allowed to solidify, before adding a cell-agar layer. The agar layers were topped up with appropriate media, DOX-containing media for experiments involving inducible shRNA. Culture media was refreshed every 72 h, and following the lysis protocol, fluorescence was measured using a plate reader.

### Flow cytometry

ALDH enzymatic activity was assessed as previously described [34–36], using ALDEFLUOR (Stem Cell Technologies, Seattle, WA) according to the manufacturer’s instructions. Following ALDH staining, cells were incubated with CD133-APC antibody (BD Biosciences, Ashland, OR) at 1:20 dilution in ALDEFLUOR buffer for 25 min on ice, protected from light. Cells were washed in PBS and resuspended in 400 µl PBS for analysis on a BD FACSVerse cell analyzer (BD Biosciences, Franklin Lakes, NJ). Cell death was assessed by Annexin V (640,905, Biolegend, San Diego, CA) and propidium iodide (PI) (R37169, Thermo Scientific, Waltham, MA) staining on cells treated as indicated, as previously described [35, 36].

### Total collagen assay

Primary human ovarian fibroblasts were plated at 1 × 10^5^ cells/well in 6-well companion plates (353,502, Falcon, Corning, NY) and cultured overnight in 2.4 mL fibroblast culture medium. The following day, 3 × 10^5^ OV90 or ACI23 cells in 1.2 mL culture media were added to cell culture inserts (0.4 µm pore size, 353,090, Falcon, Corning, NY), placed on top of the wells containing the fibroblasts and co-cultured for 3 days. The upper chambers containing OV90, ACI23, HEYA8 or SKOV3 cells were then discarded, and the Total Collagen of the primary human ovarian fibroblasts was measured using Sirius Red Total Collagen Detection Plate kit (#9026P, Chondrex, Woodinville, WA) according to the manufacturer’s instructions.

### Cytokine array and ELISA

Cytokine analysis was performed on cell culture supernatants using LEGENDplex™ HU Essential Immune Response Panel (740,930, Biolegend, San Diego, CA) according to the manufacturer’s instructions [48]. ELISAs for IL-6 (QK206, R&D Systems, Inc. Minneapolis, MN) and IL-8 (D8000C, R&D Systems, Inc. Minneapolis, MN) were performed on cell culture supernatants according to the manufacturer’s instructions.

### Quantitative real-time PCR (qRT-PCR)

Total RNA of mesothelial cells from co-culture was extracted using RNeasy Mini Kit according to the manufacturer’s instructions (74,106, Qiagen, Mansfield, MA). Total RNA was extracted from frozen xenograft tumors using TRI Reagent (AM9738, Thermo Scientific, Waltham, MA) according to the manufacturer’s instructions, immediately following overnight thawing in RNAlater™-ICE Frozen Tissue Transition Solution (AM7030, Thermo Scientific, Waltham, MA). RNA was converted to cDNA using High-Capacity cDNA Reverse Transcription Kit according to the manufacturer’s instructions (4,368,814, Applied Biosystems, Thermo Scientific, Waltham, MA). TaqMan™ Array Human Extracellular Matrix & Adhesion Molecules (4,414,133, Applied Biosystems, Thermo Scientific, Waltham, MA) and TaqMan™ Gene Expression Assays for hMMP1 (Hs00899658_m1), hFN1 (Hs01549976_m1), hLAMA3 (Hs00165042_m1), hVCAN (Hs00171642_m1), hTGFB1 (Hs00171257_m1), hTIMP3 (Hs00165949_m1), hTNC (Hs01115665_m1), hCOL1A1 (Hs00164004_m1), hCDH1(Hs01023895_m1), hIL6 (Hs00174131_m1), hIL-8 (CXCL8) (Hs00174103_m1), hCCL2 (Hs00234140_m1),), mNos2 (Mm00440502_m1) were used with TaqMan™ Fast Advanced Master Mix (4,444,963, Applied Biosystems, Thermo Scientific, Waltham, MA) and qRT-PCR was performed using ViiA 7 System. The comparative threshold cycle (Ct) method was used to calculate the relative gene expression and target genes values were normalized to the expression of the endogenous reference gene.

### In vivo studies

All animal studies were approved by the NCI Animal Care and Use Committee, IACUC Number MOB-025–1. Intra-bursal xenografts were generated by injection of 0.5 × 10^5^ cells in 5 μL PBS into the right ovarian bursa of 8-week-old female athymic Nu/Nu mice. For controls, 5 μL PBS was injected into the left ovarian bursa of each mouse. For tumor burden and histology studies, both ovarian bursa were injected with 2.5 × 10^5^ cells. Mice injected with OV90 cells containing the DOX-inducible shRNA were fed DOX chow (200 mg/kg, S3888, Bio-Serv, Flemington, NJ) for the duration of the study. For tumor regression studies, both ovarian bursa were injected with 2.5 × 10^5^ cells and mice were fed DOX chow (200 mg/kg) 7 days after injection and for the duration of the study. The animals were monitored for health and survival in days was recorded as mice met NIH Animal Care and Use Committee-approved humane criteria for euthanasia.

### Statistical analysis

In vitro assays were performed in triplicate on three independent occasions and were analyzed with t-tests or one-way ANOVA with post-tests where applicable. Results are presented as mean ± SEM with p values ≤ 0.05 considered significant. Kaplan–Meier analysis was used to analyze overall survival and progression-free survival for IHC analyses, and Mantel-Cox log-rank was used to compare groups. Statistical analyses were performed using Prism 8.0 software (GraphPad, San Diego, CA, USA).

## Results

### Identification of UGDH as a functional target in EOC spheroids

Previously we studied EOC TICs and defined characteristics that promote survival such as enhanced drug metabolism and oxidative stress management and identified drugs targeting TICs that could prevent relapse in vitro and in vivo [36]. Here, we sought to identify novel targets that functionally regulate EOC TICs and performed a whole-genome siRNA functional screen for targets that preferentially reduced viability of EOC TICs. We used the TIC-enriching spheroid culture conditions that we described previously [49] compared to adherent culture. We chose the OV90 cell line as it is *TP53* mutant, homologous recombination repair proficient, BRCA wild-type and resistant to platinum and PARP inhibitors [50, 51].

**Fig. 1:**
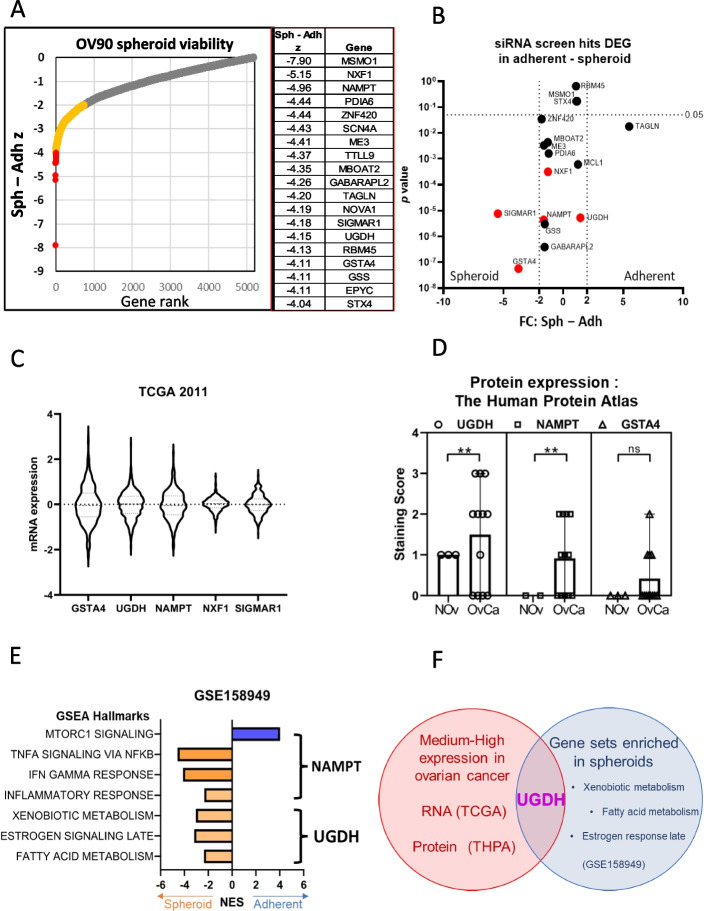
Identification of targets to inhibit the growth and survival of ovarian cancer TICs. **A** The top 20 genes identified from an siRNA functional screen that were critical spheroid viability compared to adherent cells using the Z score to compare viability. **B** RNA-seq data of OV90 cells cultured as spheroids or cultured adherently from GEO accession number GSE158949. Candidate genes were graphed for gene expression on the x axis and *p*-value on the y axis. **C** mRNA expression of five candidate genes in Ovarian Serous Cystadenocarcinoma from the Cancer Genome Atlas (TCGA). **D** Quantification of protein expression of 3 candidate genes in normal ovarian tissue and ovarian carcinomas from the Human Protein Atlas (THPA). ** *p* <0.01.  **E** Normalized Enrichment Scores (NES) for hallmarks UGDH and NAMPT were included in from GEO accession number GSE158949, comparing ovarian cancer spheroids and adherent cells by gene set enrichment analysis (GSEA). **F** Venn diagram to summarize identified target, UGDH

OV90 adherent cells or spheroids were transfected with at least 2 siRNAs per gene and viability was measured after 96 h. Using the *Z*-score of the viability of spheroids minus adherent cells, we ranked the genes that reduced spheroid viability compared to adherent, with the top 20 highlighted (Fig. 1A, Supplementary Data 1). To further refine the candidate genes, we examined their expression using RNAseq, in OV90 adherent and spheroid cells and plotted the *p*-value and fold change for spheroid compared to adherent values (Fig. 1B). Five genes with significant *p*-values (< 0.05) and enhanced or consistent expression in the OV90 cells were investigated for mRNA expression in ovarian cancer using data from The Cancer Genome Atlas (TCGA) [52] (Fig. 1C). Interestingly, three of these genes: Glutathione transferase α4 (GSTA4), Nicotinamide phosphoribosyltransferase (NAMPT) and UDP-glucose dehydrogenase (UGDH) are enzymes with roles in metabolism and detoxification which we had previously shown to be targetable pathways in TIC spheroids [36]. We also examined their protein expression in the Human Protein Atlas ([53], proteinatlas.org), and all three had low or no expression in stromal cells from normal ovarian tissue (*n* = 3), but UGDH and NAMPT expression was significantly increased in ovarian cystadenocarcinoma tissues of mucinous, endometrioid and serous histotypes (*n* = 12) (Fig. 1D). GSTA4 is a member of the Phase II detoxifying enzyme superfamily and is associated with liver cancer progression [54]. NAMPT regulates intracellular nicotinamide adenine dinucleotide (NAD) levels and cellular metabolism [55]. UGDH oxidizes nucleotide sugars to produce the subunits of hyaluronan [56], an important extracellular matrix signaling molecule that is dysregulated in EOC. We previously compared RNAseq data of ovarian cancer spheroid and adherent cells by Gene Set Enrichment Analysis (GSEA) which uses specific and well-defined biological processes to classify hallmarks [57]. From the RNAseq data, we previously showed that certain hallmarks were enriched in spheroids compared to adherent cells [36]. NAMPT is included in 4 of the hallmark gene sets, 3 of which were enriched in spheroids and one in adherent cells, and UGDH is included in 3 of the hallmark gene sets, all of which were enriched in spheroids (Fig. 1E). In this study, we chose to pursue UGDH due to its medium–high expression in EOC compared to normal ovarian tissue, its inclusion in GSEA hallmarks that were enriched in spheroids (Fig. 1F) and its reported roles in promoting cancer progression [19–21, 25–28].

### UGDH expression in epithelial ovarian cancer histotypes

EOC is a broad description for epithelial malignancies of the ovary and fallopian tube [58]. There are 5 main histotypes of EOC: clear cell, mucinous, endometrioid, high-grade serous (HGS) and low-grade serous. These subtypes differ histologically, but also in incidence, disease progression, chemotherapy response and prognosis [58]. UGDH was previously detected in mucinous adenocarcinoma and clear cell ovarian cancer tissues by immunohistochemistry (IHC) analysis and was not detected in normal adjacent tissue [28]. Here, we sought to characterize UGDH expression in curated tissue microarrays of HGS, endometrioid, mucinous and clear cell EOC histotypes and determine whether UGDH expression was prognostic.

**Fig. 2:**
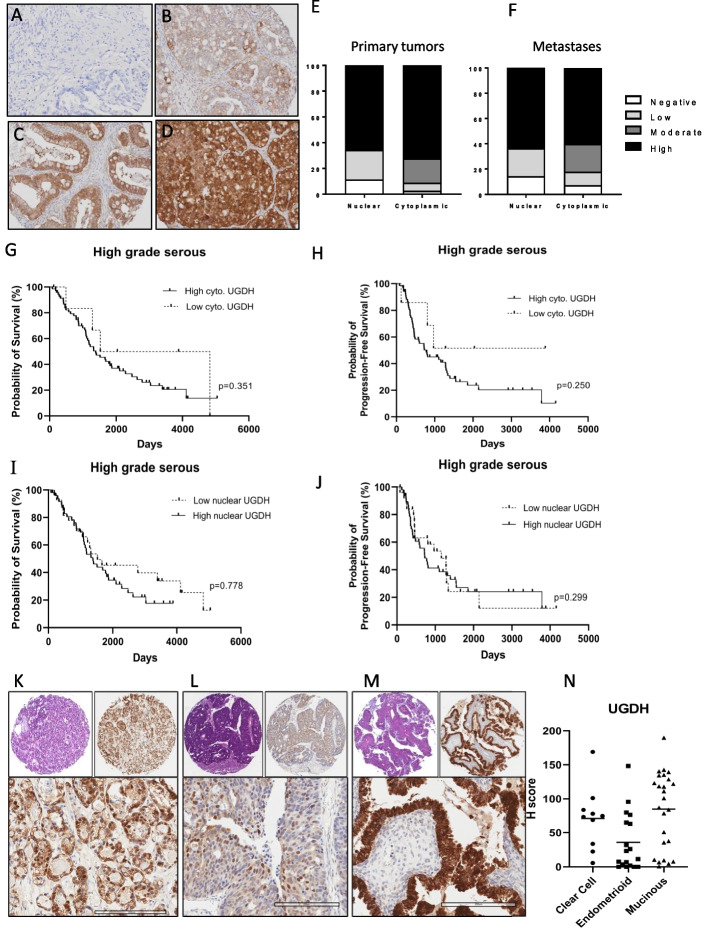
UGDH expression in ovarian cancer histotypes. Representative images of UGDH expression in high grade serous ovarian cancers that were scored as (**A**) Negative, **B** Low, **C** Moderate or (**D**) Strong, for both cytoplasmic and nuclear localization. **E** Proportions of staining scores for cytoplasmic and nuclear expression of UGDH in primary tumors and (**F**) metastases. **G** Survival analysis of HGS cancers comparing low versus high cytoplasmic UGDH. **H** Survival analysis of HGS cancers comparing low versus high nuclear UGDH. **I** Progression-free survival analysis of HGS cancers comparing low versus high cytoplasmic UGDH. **J** Progression-free survival analysis of HGS cancers comparing low versus high nuclear UGDH. **K** Clear cell, Stage 3C, top left panel H&E in 4X, top right panel IHC in 4X, lower panel IHC 20x, **L** Endometrioid, stage 3C, top left panel H&E in 4X, top right panel IHC in 4X, lower panel IHC 20x. **M** Mucinous Stage 3C top left panel H&E in 4X, top right panel IHC in 4X, lower panel IHC 20x. **N** Expression of UGDH expressed as H-score. Scale bar is 200 µm

The HGS TMA contained 96 patient tissues, sampled from primary and metastatic sites, and UGDH expression was scored based on previous methods [30, 59] as negative, weak, moderate, and strong for both cytoplasmic and nuclear localization (Fig. 2A-D). There was a high percentage of positive staining detected overall with only 2.5% of cases being scored as negative for cytoplasmic staining and 11.4% negative for nuclear staining. The distribution of staining intensity and localization in primary and metastatic sites were similar (Fig. 2E, F). Correlative analyses were performed on UGDH expression and clinicopathological data, for overall survival and progression-free survival analysis (Supplementary Table 1). We found that subcellular localization was not prognostic for HGS (Fig. 2G-J). Nuclear expression in HGS was not associated with progression or survival as it was reported for lung adenocarcinoma [19]. Although cytoplasmic UGDH expression was associated with survival outcome (Supplementary Table 1), it was not prognostic for overall or progression-free survival (Fig. 2G-J). Further investigation with more samples to increase the numbers of HGS cases that were negative for or weakly expressed UGDH may reveal a prognostic effect.

For the mucinous, endometrioid and clear cell EOC cases, scoring of UGDH expression was performed using H score which measures the intensity and proportion of staining [31], (Fig. 2K-M). There was abundant UGDH expression in these subtypes, with the highest median expression seen in the mucinous subtype (Fig. 2N). UGDH localization did not have prognostic value in these subtypes. Using the median H score, cases were classified as having higher or lower than the median expression for clinicopathological analyses [60], (Supplementary Table 2). In endometrioid EOC cancers, UGDH expression was associated with stage, where UGDH expression was higher at lower disease stages while expression decreased at higher stage. The number of cases were not sufficient to robustly show differences in overall survival or progression-free survival based on UDGH expression or localization for these subtypes. Of note, most of the cases in the clear cell and mucinous subtypes were International Federation of Gynecology and Obstetrics (FIGO) stage 1 and 2 cancers [61], which typically have a higher survival and lower recurrence rate [62].

### High UGDH expression is associated with poor prognosis in the C1/Mesenchymal molecular subtype

In prior work, Tothill et. al profiled the gene expression of 285 serous and endometrioid ovarian, fallopian tube, and peritoneal cancers, as well as a smaller number of low-grade, low malignant potential tumors. The tumors were categorized into six molecular subtypes [29]. The high-grade cancers clustered into subtypes designated C1, C2, C4 and C5, while the C3 and C6 subtypes clustered the low grade, early stage and low malignant potential tumors [29]. The subtypes were characterized by gene expression, histology, immune infiltration, stromal desmoplasia, and prognosis, which showed that the C1 and C5 subtypes correlated with a poorer overall and progression-free survival compared to the other subtypes [29]. Molecular subtypes for EOC were later characterized independently by the TCGA consortium, and were characterized as Mesenchymal, Immunoreactive, Differentiated and Proliferative [52]. Tumors of the C1/Mesenchymal subtype can be identified by profound desmoplasia and myofibroblast activity, a strong enrichment of ECM remodeling genes and poor survival [63]. The C2/Immunoreactive subtype has high levels of intratumoral T-cell infiltration and an adaptive immune response gene signature [29]. C4/Differentiated tumors express higher levels of ovarian tumor markers such as MUC16 (CA125) compared to the other subtypes, sharing features of borderline serous tumors [29, 63]. The C5/Proliferative subtype shares some mesenchymal features, low inflammatory cell infiltration in tumors and a poor survival outcome but also activation of oncogenic signaling through WNT/β-catenin and homeobox genes [29, 52]. Here, we examined UGDH expression in the same TMA used by Tothill et., al and describe the subtypes using both the Tothill and TCGA designations: C1/Mesenchymal (C1/MES), C2/Immunoreactive (C2/IMR), C4/Differentiated (C4/DIF), C5/Proliferative (C5/PRO).

**Fig. 3:**
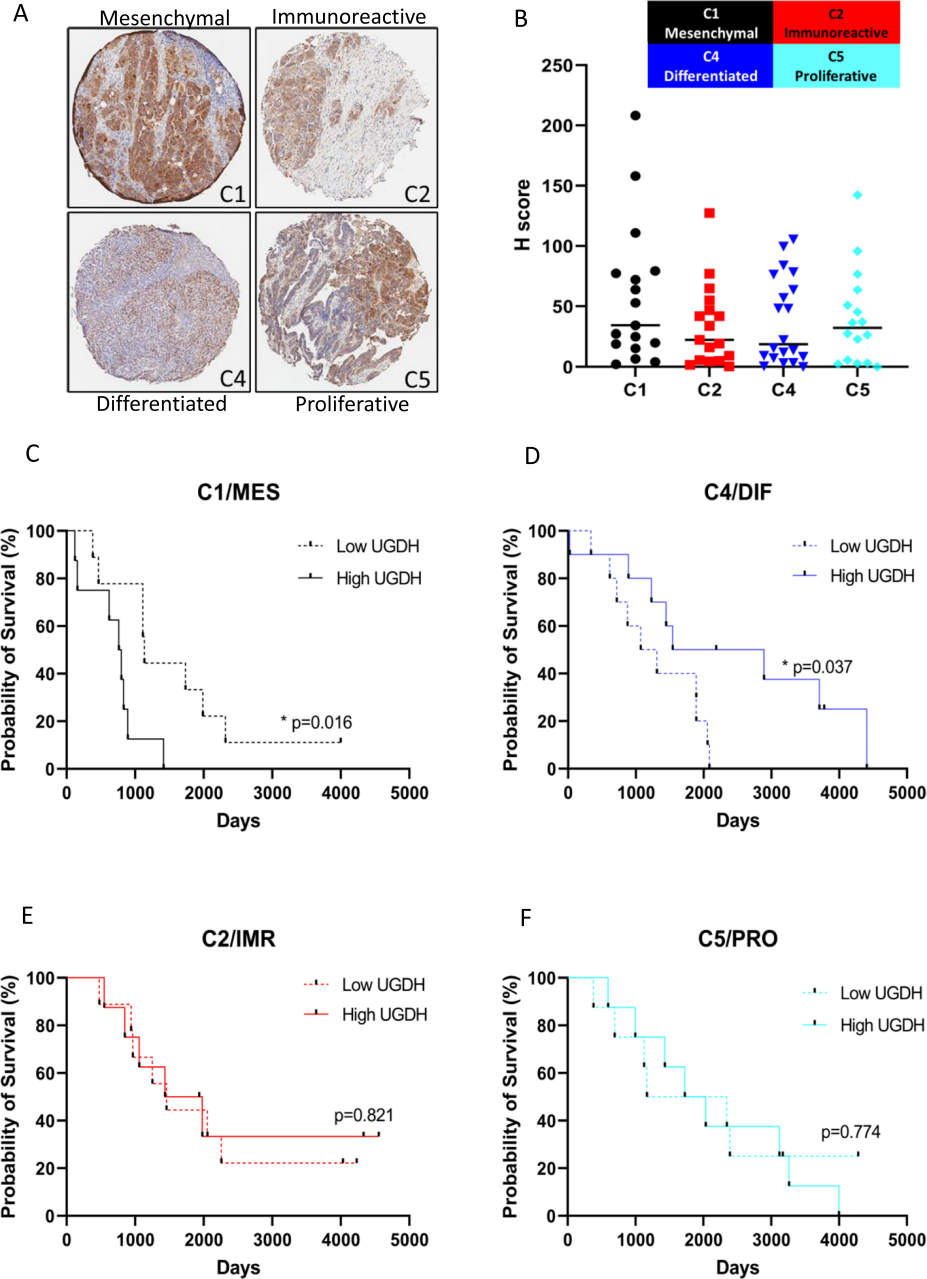
UGDH expression in molecular subtypes of high grade epithelial ovarian cancers. **A** Representative images of UGDH expression in TMA cores from molecular subtypes C1, C2, C3 and C4 at 4X magnification. **B** Expression of UGDH expressed as H-score. **C** Survival analysis of C1 subtype comparing low versus high UGDH H-score (above or below the median). **D** Survival analysis of C4 subtype comparing low versus high UGDH H-score (above or below the median). **E** Survival analysis of C2 subtype comparing low versus high UGDH H-score (above or below the median). **F** Survival analysis of C5 subtype comparing low versus high UGDH H-score (above or below the median)

UGDH expression was highest in the C1/MES subtype, followed by the C5/PRO subtype (Fig. 3A, B). The C1/MES subtype has the poorest prognosis of the subtypes [29] and high UGDH expression correlated with shorter overall survival (Fig. 3C), but not progression-free survival (Supplementary Table 3). The C1/MES subtype was classified by a high stromal signature, with gene expression increases in ECM proteins, proteoglycans and histologically a high level of desmoplasia [29]. Interestingly, the C4/DIF subtype that is classified as a low stromal signature but increased immune infiltration, showed the opposite prognostic result for UGDH expression: low UGDH expression was associated with a significantly poorer overall survival (Fig. 3D), and progression-free survival (Supplementary Table 3). The C2/IMR and C5/PRO subtypes did not show significant correlations of UGDH expression with prognosis (Fig. 3E, F). We also examined nuclear and cytoplasmic localization of UGDH in the molecular subtypes for prognostic value (Supplementary Table 3). Cytoplasmic and nuclear expression was similar among the cases, in that the cases with high UGDH expression had both high cytoplasmic and nuclear expression (Supplementary Fig. 1). In the C4/DIF subtype however, the prognostic effect of high UGDH expression tended to be related to cytoplasmic rather than nuclear expression (Supplementary Table 3).

### UGDH expression in cell lines clustered by molecular subtyping analysis

**Fig. 4:**
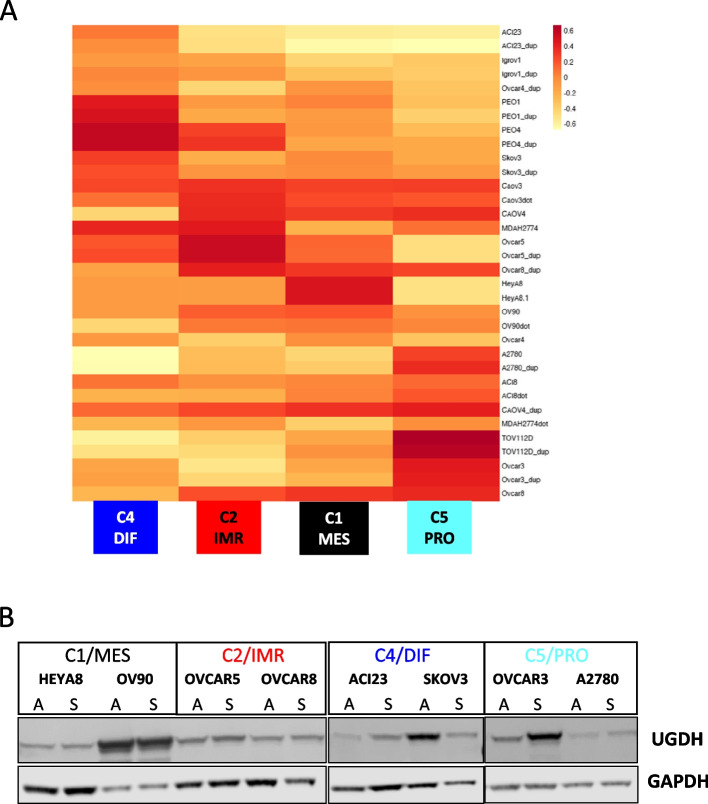
Ovarian cancer cell lines clustered into molecular subtypes examined for UGDH expression. **A** Heatmap of cell lines aligned with molecular subtypes. **B** Expression of UGDH in cell lines in adherent (**A**) and spheroid (S) culture conditions by Western blot analysis

To model the molecular subtypes in vitro we classified ovarian cancer cell lines into the molecular subtypes originally annotated by two independent datasets of primary cancer specimens [29, 52]. Molecular subtyping of ovarian cancer cell lines was previously reported, using a different clustering method that classified novel molecular subtypes [64]. However, we sought to identify cell lines to represent the subgroups in which we identified prognostic implications for UGDH. Therefore, we performed k-means clustering analysis of 17 EOC cell lines with biological duplicates, grown in adherent conditions to retain integrin, adhesion and ECM related genes for classification into molecular subtypes C1/MES, C2/IMR, C4/DIF, and C5/PRO (Fig. 4A) [65]. Two representative cell lines for each subtype were examined for expression of UGDH from both adherent and TIC spheroid culture conditions by Western blot analysis (Fig. 4B). UGDH expression was highest in OV90 (C1/MES) in both culture conditions, and notably OVCAR3 in the C5/PRO subtype showed elevated expression in the TIC spheroid culture condition. UGDH expression in the cell lines did resemble the finding of the IHC performed on patient samples of the molecular subtypes, where the C1 subtype tumors had the highest median H-score for UGDH, followed by the C5/PRO subtype and lower expression in the C2/IMR and C4/DIF subtypes. From this analysis, we used OV90 and HEYA8 to represent the C1/MES subtype, in which high UGDH expression correlated with poorer survival, and ACI23 and SKOV3 to represent the C4/DIF subtype, in which low UGDH expression correlated with shorter survival.

### Spheroid viability and cell morphology is affected by UGDH expression

**Fig. 5:**
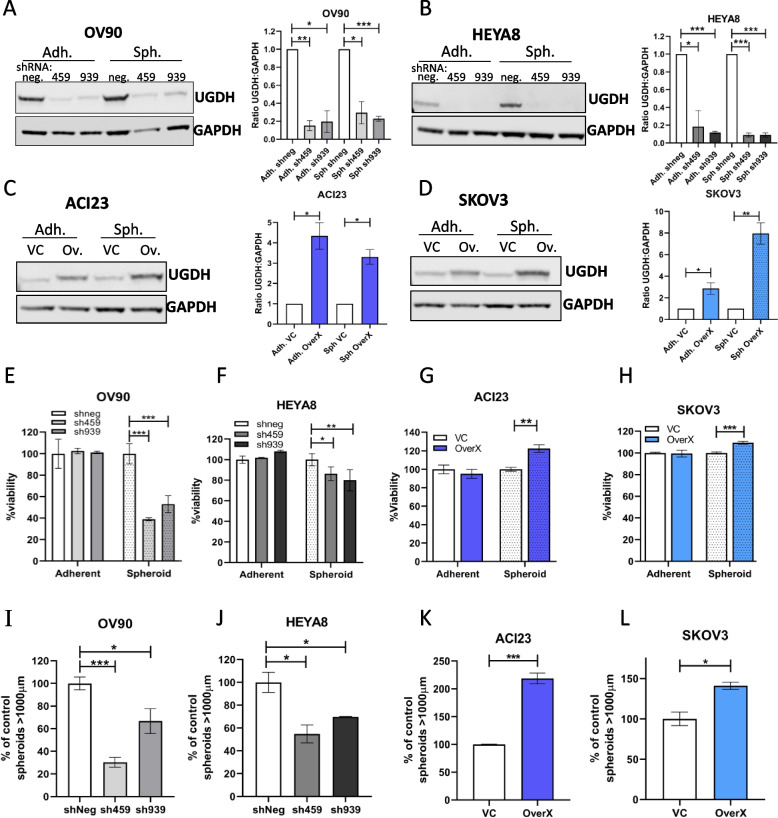
Effects of UGDH knockdown in C1/MES cell lines, and over-expression in C4/DIF cell lines on adherent and spheroid growth in vitro. Western blot and densitometry analysis of UGDH expression in C1/MES cell lines (**A**) OV90 cells, and (**B**) HEYA8 cells, with DOX-inducible negative control shRNA (shneg) or DOX-inducible shRNA targeting UGDH (sh459, sh939) after 3 days of DOX induction in adherent (Adh.) or spheroid (Sph.) culture conditions. Western blot and densitometry analysis of UGDH expression in C4/DIF cell lines: **C** ACI23 cells, and (**D**) SKOV3 cells with stably expressed vector control (VC) or UGDH (Ov, OverX) grown for 3 days in, and adherent (Adh.) or spheroid (Sph.) culture conditions. Viability of C1/MES cells grown in adherent or spheroid conditions with control (shneg) and UGDH-knockdown (sh459,sh939): **E** OV90, **F** HEYA8. Viability of C4/DIF cells grown in adherent or spheroid conditions with control (VC) and UGDH overexpression (OverX): **G** ACI23, **H** SKOV3. Sphere forming capacity of C1/MES cell lines with control (shneg) or UGDH knockdown (sh459, sh939) in (**I**) OV90 and (**J**) HEYA8. Sphere forming capacity of C4/DIF cell lines with control (VC) or UGDH overexpression (OverX): **K** ACI23, **L** SKOV3. n.s non-significant, *p < 0.05, **p < 0.01, ***p < 0.001

In the C1/MES molecular subtype, high UGDH expression was associated with poorer prognosis whereas high UGDH expression was associated with improved prognosis in the C4/DIF subtype. Therefore, in comparing the effect of UGDH expression in these subtypes, we silenced UGDH expression in C1/MES cell lines OV90 and HEYA8 using inducible shRNA, and over-expressed UGDH in C4/DIF cell lines ACI23 and SKOV3. Western blot analysis and densitometry showed efficient silencing of UGDH was induced in OV90 and HEYA8 cells (sh459, sh939) after 3 days of doxycycline (DOX) compared to control (shneg) in both adherent and spheroid culture conditions (Fig. 5A, B). Over-expression of UGDH was achieved in ACI23 and SKOV3 cells (Ov.) compared to control (VC) in both adherent and spheroid culture conditions (Fig. 5C, D). UGDH knockdown in OV90 changed the morphology of adherent cultures to appear more epithelial and ‘cobblestone’ like (Supplementary Fig. 2A); in contrast, overexpression of UGDH in ACI23 did not significantly change their appearance (Supplementary Fig. 2B). It was previously reported that UGDH mediates metastasis and epithelial-mesenchymal transition (EMT) in lung cancer [20]. Therefore, we examined the expression of EMT markers Vimentin and E-cadherin in OV90 cells with UGDH silencing. Consistent with the previously reported findings in lung cancer cells, UGDH knockdown increased E-cadherin expression in OV90 cells (Supplementary Fig. 2C, D).

To validate the findings from the initial siRNA screen of OV90 cells identifying UGDH as a potential target against TICs, cell viability in adherent and spheroid culture conditions was examined. In adherent conditions, alteration of UGDH expression did not significantly affect viability of either cell line. However, induction of knockdown in formed OV90 spheroids significantly reduced viability by 48–60% (Fig. 5E), and modestly reduced HEYA8 spheroid viability (Fig. 5F), confirming the effect observed in the siRNA screen. Overexpression of UGDH increased spheroid viability of ACI23 and SKOV3 cells by 41% and 18% respectively, compared to vector controls (Fig. 5G, H). These data are summarized in Supplementary Table 4, and show that adherent cell morphology, but not viability was altered by UGDH silencing in OV90 cells. Spheroid viability was greatly reduced when UGDH was silenced in the C1/MES subtype cell lines but was enhanced when UGDH was overexpressed in the C4/DIF subtype cell lines.

### UGDH silencing in C1/MES, and over-expression in C4/DIF, reduces TICs in vitro

**Fig. 6:**
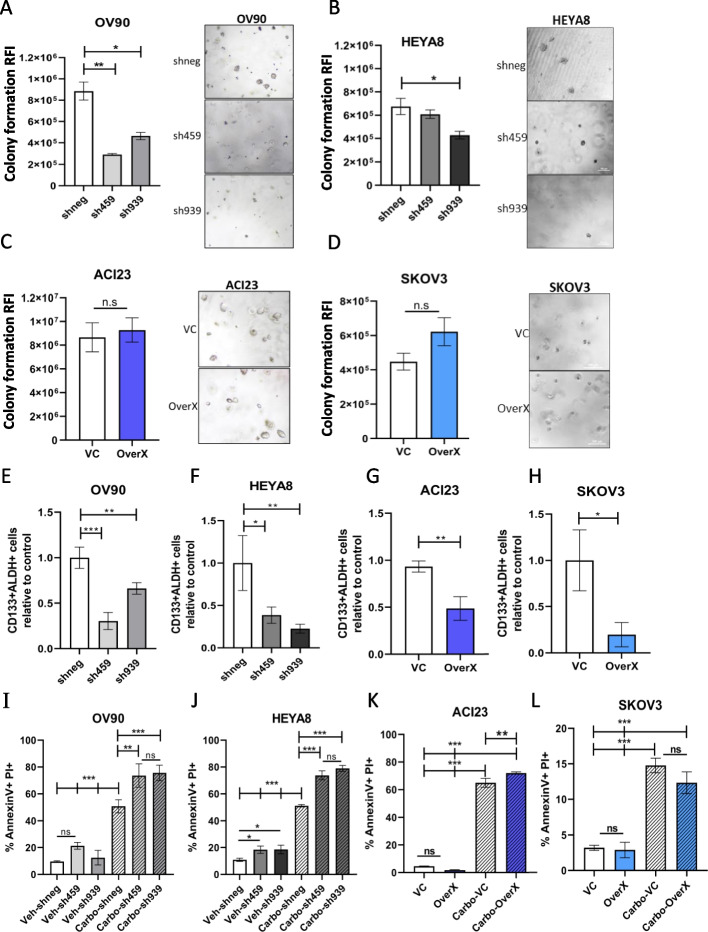
Effects of UGDH knockdown in C1/MES cell lines, and over-expression in C4/DIF cell lines on TIC populations and functions in vitro. Colony forming capacity of C1/MES cell lines with UGDH knockdown (sh459, sh939) compared to control (shneg) in: **A** OV90, **B** HEYA8. Colony-forming capacity in of C4/DIF cell lines with control (VC) and UGDH overexpression (OverX): **C** ACI23, **D** SKOV3. Quantification of the proportion of CD133 + ALDH + cells in C1/MES cell lines with UGDH knockdown (sh459, sh939) compared to control (shneg) in: **E** OV90 and (**F**) HEYA8. Quantification of the proportion of CD133 + ALDH + population in C4/DIF cell lines with control (VC) and UGDH overexpression (OverX) in (**G**) ACI23, **H** SKOV3. Analysis of cell death by AnnexinV and PI double positive cell population from in vitro relapse model in spheroids generated from viable cells collected after 48 h of carboplatin treatment followed by induction of UGDH silencing (sh459, sh939) compared to control (shneg) in C1/MES cells (**I**) OV90, **J** HEYA8 or with UGDH overexpression (OverX) or control (VC) in C4/DIF cell lines in (**K**) ACI23, **L** SKOV3. n.s non-significant, *p < 0.05, **p < 0.01, ***p < 0.001

The spheroid culture condition enriches for the TIC population in ovarian cancer cell lines, which causes enhanced tumor growth in mouse models and promotes relapse [34, 36, 49]. Therefore, we examined whether targeting UGDH could affect the features of TICs including spheroid formation, colony formation, expression of stem cell markers, and relapse in vitro. The effect of modulating UGDH expression was examined on spheroid formation where UGDH silencing was induced from the time of plating. In C1/MES OV90 and HEYA8 cells, UGDH silencing significantly reduced the spheroid forming capacity compared to negative controls, by 35–70% and 30–45%, respectively (Fig. 5I, J). Overexpression of UGDH in C4/DIF ACI23 and SKOV3 cells however, increased the number of spheres compared to the vector control, by 118% and 41%, respectively (Fig. 5K, L). The colony forming capacity of C1/MES cells OV90 and HEYA8 was significantly reduced by UGDH knockdown compared to negative controls (Fig. 6A, B), but overexpression in C4/DIF cells caused no significant difference (Fig. 6C, D). We and others have shown that the CD133 + /ALDH ^high^ cell population are TICs [34, 49, 66]. Examining these markers in spheroid cultures of cell lines with altered UGDH expression revealed that silencing in C1/MES lines OV90 and HEYA8 cells (Fig. 6E, F), and overexpression in C4/DIF lines ACI23 and SKOV3 (Fig. 6G, H), caused a significant reduction in this population compared to controls. The same effect caused by opposing expression of UGDH in the cell lines may be explained by different mechanisms. In OV90 and HEYA8 cells, the reduction of viability caused by UGDH knockdown in spheroids may explain the overall reduction in CD133 + /ALDH ^high^ cells. And in ACI23 and SKOV3 cells, overexpression of UGDH may out-compete ALDH for NAD + substrate, as both are dependent on this for activity [16, 67], thus causing reduced ALDH activity to be observed. Finally, we used our previously reported in vitro relapse model [35, 36] to directly assess the potential for spheroids with altered UGDH to promote growth and persist after chemotherapy. The cell lines were grown adherently for 48 h and treated with a sub-lethal dose of carboplatin or vehicle; the viable populations remaining after treatment were then cultured in TIC-enriching spheroid conditions and assessed for cell death. Knockdown of UGDH in OV90 and HEYA8 spheroids after carboplatin treatment significantly increased cell death, compared to the negative control (Fig. 6I, J). Significantly increased cell death was also observed in ACI23 spheroids generated after carboplatin treatment overexpressing UGDH compared to the vehicle control (Fig. 6K), although this was not replicated in the SKOV3 (Fig. 6L). These data are summarized in Supplementary Table 4, and indicate that differential UGDH expression is important for the formation and composition of the spheroids and the TIC population that drives recurrence. UGDH was highly expressed in EOC spheroids in the C1/MES subtype which is classified by high stromal activity and poor prognosis, and knockdown in this subtype reduced spheroid and TIC function suggesting UGDH promotes survival of an aggressive population of cells. Contrastingly, high expression of UGDH in C4/DIF subtype was associated with improved prognosis, and overexpression in cell lines increased spheroid formation but not TIC-related functions, suggesting it has a role in proliferation but not stemness in this subtype.

### UGDH expression in EOC spheroids alters cytokine secretion, and differentially influences cells in the tumor microenvironment

**Fig. 7:**
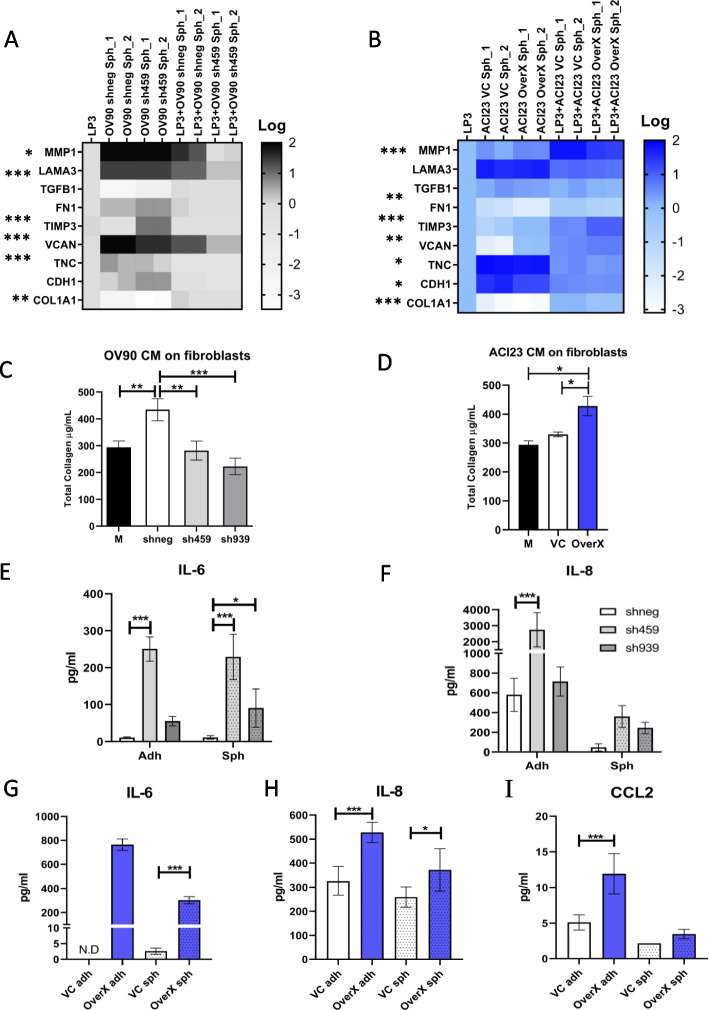
Changes to UGDH expression in spheroids alters cells and cytokines of the tumor microenvironment in vitro. In (**A**) and (**B**), spheroids were generated, and knockdown induced with DOX before co-culture with LP3 mesothelial adherent monolayers for 24 h and gene expression of co-cultures was measured by qRT-PCR. **A** Heatmap of expression of genes altered in OV90 control spheroids (shneg) compared to OV90 spheroids with UGDH knocked down (sh459), and in co-culture with LP3 cells, relative to LP3 cells alone. **B** Heatmap of expression of genes altered in ACI23 control spheroids (VC) compared to ACI23 spheroids with UGDH overexpression (OverX), and in co-culture with LP3 cells, relative to LP3 cells alone. Total collagen content of fibroblasts was measured after 3 days culture in conditioned medium from: **C** OV90 control cells (shneg) or OV90 cells with UGDH knocked down (sh459. sh939) compared to OV90 culture medium (M), **D** ACI23 cells with UGDH overexpression (OverX) or vector control (VC) compared to ACI23 culture medium (M). Expression of (**E**) IL-6 (**F**) IL-8 in supernatant from OV90 adherent cells or spheroids with UGDH knockdown (sh459, sh939) compared to controls (shneg). Expression of (**G**) IL-6 (**H**) IL-8 (**I**) CCL2 in supernatant from ACI23 adherent cells or spheroids with UGDH overexpression (OverX) compared to controls (VC). N.D not detected, *p < 0.05, **p < 0.01, ***p < 0.001

In comparing the C1/MES and C4/DIF molecular subtypes, stromal response was the major histological difference between these groups. Therefore, we asked if UGDH expression in tumors also contributed to the microenvironment. In the peritoneum, mesothelial cells are the predominant stromal cell type that form a protective barrier for tissues, but also contribute to tissue repair, regulate inflammation in the microenvironment by cytokine secretion and can support adhesion and invasion of metastatic cells [68]. Therefore, we next examined an in vitro model of the peritoneal stroma of EOC by co-culturing mesothelial cell line LP3 with EOC spheroids with altered UGDH expression. We assessed gene expression in of LP3 cells in co-culture with UGDH-altered spheroids, as well as spheroids alone by qRT-PCR, and compared the differences as relative to LP3 cells alone. When UGDH was knocked down in the OV90 spheroids representing the C1/MES subtype, there was a decrease in the expression of ECM components VCAN and TNC, and increased expression of metalloprotease inhibitor TIMP3, and cell–matrix interacting proteins FN1 and CDH1 (Fig. 7A). When these spheroids were co-cultured with LP3, there was a further decrease in VCAN expression, as well as a decrease in matrix remodeling enzyme MMP1 and ECM interacting protein LAMA3 expression compared to LP3 alone. These changes suggest that UGDH knockdown on the C1/MES spheroids causes a decrease in extracellular matrix remodeling and invasive potential due to decreased matrix protease and ECM component expression. We also examined the expression of the same markers in co-cultures of the ACI23 spheroids representing the C4/DIF subtype, with overexpression of UGDH. The overexpression in this subtype replicated some of the effects of knockdown in the C1/MES spheroids, where MMP1 expression was decreased, and TIMP3 expression was increased when UGDH was overexpressed in the spheroids and when in co-culture with LP3 (Fig. 7B). However, VCAN expression increased in the overexpressing spheroids and in co-culture. Other changes in this subtype with overexpressed UGDH included reduced COL1A1, FN1 and TGFB expression in co-cultures compared to LP3 alone, as well as reduced CDH1 in spheroids. This suggests that the microenvironment of the C4/DIF subtype may become more desmoplastic, through altered activity of stromal cells when UGDH is overexpressed.

We next examined whether altered expression of UGDH in cancer cells could indirectly influence cells in the TME, using a co-culture of EOC cells and fibroblasts that allowed media exchange but not cell contact between fibroblasts and EOC cells. Normal human ovarian fibroblasts in co-culture with OV90 cells had increased total collagen production compared to media-only control, but when UGDH was silenced in C1/MES cells OV90 or HEYA8, co-cultured fibroblast’s total collagen was significantly reduced (Fig. 7C, Supplementary Fig. 3A, respectively). When fibroblasts were co-cultured with ACI23 cells, their total collagen content did not differ to the controls, fibroblasts cultured with ACI23 cell media only. However, when UGDH was overexpressed in C4/DIF cells ACI23 or SKOV3, fibroblasts in co-culture had significantly increased total collagen (Fig. 7D, Supplementary Fig. 3B, respectively). As cytokines can be modulated by the ECM and influence the TME, we were also interested in whether UGDH expression influenced cytokine secretion in the EOC spheroids. In C1/MES cells OV90 and HEYA8, when UGDH was knocked down, IL-6 and IL-8, levels increased significantly (Fig. 7E, F, Supplementary Fig. 3C, D, respectively). In the C4/DIF ACI23 cells when UGDH was overexpressed, there was a significant increase in IL-6, IL-8, and CCL2 compared to controls (Fig. 7G, H, I). Consistent with this, C4/DIF SKOV3 spheroids overexpressing UGDH also had increased IL-6 and IL-8 (Supplementary Fig. 3E, F). These data suggest UGDH differentially influences cells in the tumor microenvironment, and regulates inflammatory cytokines in a subtype-specific manner.

### UGDH promotes fibroinflammatory changes in the stroma of tumor xenografts and silencing reduced tumor burden in vivo

**Fig. 8:**
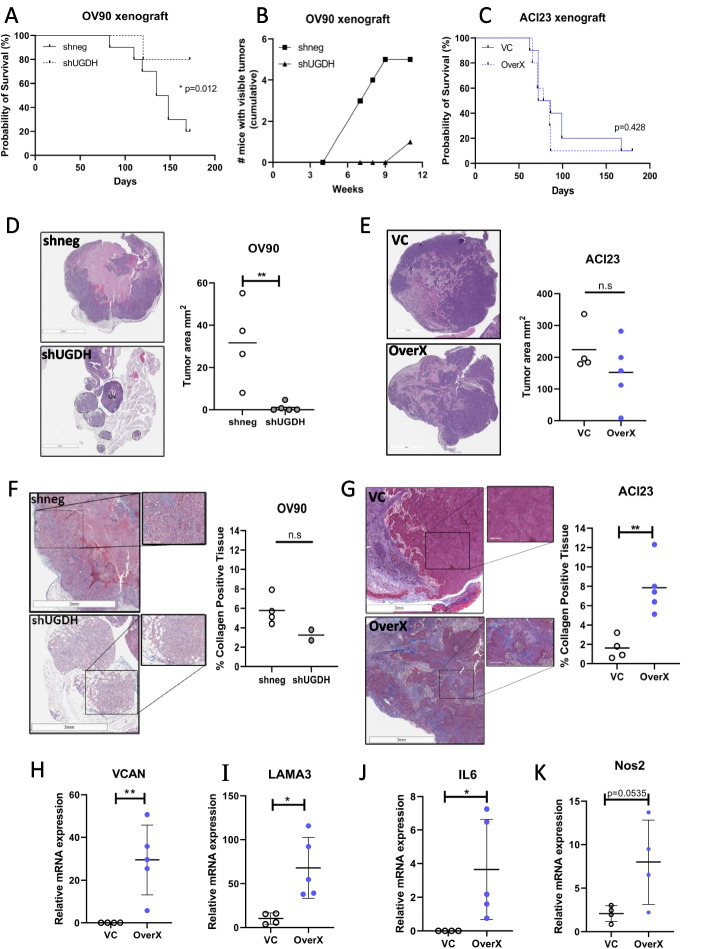
Overall survival, tumor size and histomorphology of xenografts of OV90 with UGDH knockdown, and ACI23 with UGDH overexpression. **A** Survival analysis of OV90 control (shneg) or UGDH knockdown (shUGDH) intrabursal xenografts. **B** Cumulative totals of mice with visible tumors at necropsy, at indicated timepoints. Intrabursal xenografts of OV90 control (shneg) or UGDH knockdown (shUGDH) cells were injected and allowed to establish for 7 days prior to DOX induction of shRNAs. **C** Survival analysis of ACI23 control (VC) or UGDH overexpression (OverX) intrabursal xenografts. **D** H&E images of OV90 xenografts (left) and quantification of tumor size (right). Tumor is marked by dashed lines; ovary is marked Ov. Scale bar is 3 mm. **E** H&E images of ACI23 xenografts (left) and quantification of tumor size (right). Scale bar is 3 mm. **F** Massons trichrome staining images of OV90 xenografts (left) and quantification of collagen in the tissue (right), scale bar is 3 mm for lower power image, 300 µm for inset higher power image. **G** Massons trichrome staining images of ACI23 xenografts (left) and quantification of collagen in the tissue (right), scale bar is 3 mm for lower power image, 300 µm for inset higher power image. **H** Expression of VCAN, **I** LAMA3 (**J**) IL6 and (**K**) Nos2 mRNA in ACI23 control (VC) and UGDH overexpressing (OverX) xenograft tumors. n.s non-significant,*p < 0.05, **p < 0.01

The effect of UGDH knockdown in C1/MES and overexpression in C4/DIF was tested on mouse intrabursal xenografts of OV90 and ACI23 cells, respectively. The mice were followed for overall survival to determine if the same prognostic outcome that was observed in the patients could be replicated. In the C1/MES groups, knockdown of UGDH in OV90 xenografts significantly improved survival compared to the negative control OV90 xenografts (Fig. 8A). These results replicate the prognostic results of UGDH expression in patients with EOC in the C1/MES molecular subtype. The small numbers of viable tumor from OV90 xenografts with UGDH knockdown prevented thorough assessment of effects in vivo. To test if UGDH silencing would affect the growth of established tumors, OV90 intrabursal xenografts were allowed to establish for 7 days before induction of shRNAs with DOX and mice were followed over 11 weeks. Necropsy was performed at different timepoints, and mice were inspected for tumors. Macroscopic tumors were visible in 5 of 6 OV90 control xenografts, compared to 1 of 6 UGDH knockdown xenografts (Fig. 8B). This suggests that UGDH expression is important for tumor establishment and outgrowth in the C1/MES subtype. In comparison, overexpression of UGDH in the C4/DIF ACI23 xenografts did not significantly affect survival compared to controls (Fig. 8C). The changes to gene expression of co-cultured cells in vitro also prompted investigation of the histomorphology of OV90 and ACI23 xenografts. The xenografts of ACI23 and OV90 differed greatly, with OV90 xenografts growing as multiple foci of smaller neoplastic masses in the bursa and some intratumoral hemorrhage (Fig. 8D), whereas ACI23 xenografts manifested as large, differentiated neoplasms with areas of necrosis within the ovarian bursa (Fig. 8E). Within the OV90 xenografts, UGDH knockdown significantly reduced tumor burden compared to controls (Fig. 8D). In comparison, overexpression of UGDH in the ACI23 xenografts did not significantly affect tumor size (Fig. 8E). The histomorphology of the xenografts was examined for fibrosis and collagen deposition using Masson’s trichrome stain (Fig. 8F, G). Tumors with UGDH overexpression showed enhanced collagen deposition but fibrotic stroma (Fig. 8G) and increased expression of VCAN, LAMA3 (Fig. 8H, I) and inflammatory markers IL-6 and Nos2 (Fig. 8J, K), consistent with in vitro co-culture findings. Additionally, the xenograft tumors were examined for the presence of macrophages. There was no significant difference in total immune cell infiltration by CD45 expression between the control and UGDH overexpressing tumors (Supplementary Fig. 4A, B) however tumors with UGDH overexpression showed an increased number of macrophages by Iba1 staining (Supplementary Fig. 4C, D), but the expression of polarization markers CD206 or iNOS were not significantly different between the groups (Supplementary Fig. 4E-H). These data indicate that the increased expression of UGDH in tumor cells of the C4/DIF subtype influences the TME to become fibrotic, inflammatory and attractive to macrophages.

## Discussion

The TME of EOC is a complex, immunosuppressive network of heterotypic cell types supported by ECM, cytokines and growth factors and presents a significant challenge to treatment, especially in the mesenchymal molecular subtype. Disease progression and recurrence in EOC is promoted by the TME and the survival of TICs in spheroids, which are targets for therapeutic eradication. Taking an integrative approach to identify genes essential to spheroid survival, we designed a functional whole genome siRNA screen to assess spheroid viability, and examined RNAseq data for differential gene expression in spheroids to refine candidate genes and identified the enzyme UGDH as critical to spheroids. We characterized UGDH expression in EOC and identify its roles in supporting TICs and its influence on the TME. We identified key subtype-specific differences indicating that UGDH pro-tumorigenic activity predominates in the mesenchymal subtype of HGS ovarian cancer. This has important implications for the development of therapeutic strategies in this disease.

The expression and prognostic significance of UGDH varies by EOC subtype. We examined UGDH expression in mucinous and clear cell EOC subtypes and found it was elevated compared to normal adjacent tissue. In our extensive range of EOC TMAs we also found strong expression of UGDH in high-grade serous cancers, to such a degree that it was not feasible to correlate with prognoses due to the few cases of negative staining observed. In the clear cell, endometrioid and mucinous tissues we saw a variation in expression, but this was also not indicative of prognoses in the low numbers of cases examined. More cases may provide insight into UGDH as a prognostic marker in these histotypes. We also examined the subcellular localization of UGDH in the TMAs to determine if it had a prognostic indication for EOC, similar to what was reported for lung adenocarcinoma [19]. In lung adenocarcinoma positive nuclear UGDH localization correlated with lymphatic and vascular invasion, larger tumor size, higher stage, and poor differentiation [19]. However, we did not find any correlation between UGDH localization and clinicopathological data in our samples; most samples were positive for both nuclear and cytoplasmic localization. This suggests that in EOC, the function of UGDH in promoting cancer progression is not linked to distinct nuclear or cytoplasmic roles. Moreover, the most significant prognostic indication of UGDH expression was found in the molecular subtypes of EOC. We showed that UGDH expression correlated with prognosis in the molecular subtypes C1/MES and C4/DIF which have distinct stromal phenotypes in terms of histology and immune infiltration [29]. Importantly, high UGDH expression had opposite effects in these subtypes. This finding suggests that if therapies were designed to block UGDH activity, they should be specifically directed to women with the C1/Mesenchymal molecular subtype and not the C4/Differentiated type of HGS.

The C1/MES molecular subtype was described as high stromal reactive, with extensive desmoplasia and immune infiltration within the stroma but lower intertumoral infiltration [29]. These observations suggest that the C1/MES tumor types are inflammatory but protected from intratumoral immune infiltration, suggesting an immune excluded tumor phenotype. Examining this subtype using the OV90 cell line with shRNA revealed UGDH as essential for spheroid viability, TIC viability and importantly, altered the TME in co-cultures in vitro and in xenografts. Analysis of the gene expression from co-culture of OV90 knockdown in spheroids with mesothelial cells showed decreased expression of ECM components VCAN, LAMA3 and MMP1 and increased expression of differentiation and fibrosis markers CDH1 and FN1. These changes in gene expression may be the result of altered activity of the transcription factor Wilms Tumor 1 (WT1) that predominates in mesothelial cells and regulates growth and differentiation [69]. Additionally, fibroblasts in co-culture, but not direct contract with OV90 cells, had reduced collagen when OV90 cells had UGDH knockdown. Our findings align with previous reports of the effects of UGDH knockdown in cancer. In glioblastoma cell lines, silencing of UGDH with siRNA reduced viability and migration of cancer cells in vitro and tumor growth in vivo, largely due to the reduction of ECM proteins tenascin and laminin that promote glioblastoma progression [21]. In breast cancer models, UGDH knockdown caused increased CDH1 and FN1 expression [26]. The C1/MES tumor phenotype was replicated in the OV90 xenografts, with activated inflamed stroma observed in the OV90 negative control xenografts. Additionally, in line with what was observed in patients with the C1/MES subtype, overall survival improved in mice with UGDH knockdown OV90 xenografts compared to controls. An interesting phenotype of the OV90 knockdown tumors was the significantly impaired tumor growth compared to controls. Future studies will be done to investigate whether UGDH knockdown can prevent relapse in a post-surgery, post-chemotherapy mouse model.

In contrast to the C1/MES subtype, the C4/DIF molecular subtype was described as having a low stromal response histologically and genetically, moderate immune infiltration in tumor and stroma and expression of markers of differentiation including E-cadherin, MUC16 and MUC1 [29, 52]. In this subtype, low UGDH expression was associated with a poorer prognosis. When UGDH was overexpressed in the C4/DIF cell lines, we observed increased spheroid formation capacity but a reduced TIC population, suggesting it doesn’t enhance stemness in this subtype. The low stromal activity in this subtype and low-moderate tumor immune infiltration suggests this tumor subtype is not inflamed, or is immune excluded and may represent a ‘cold tumor’. In vivo, overexpression of UGDH in ACI23 xenografts did not significantly affect tumor size or necrosis. However increased collagen deposition in tumors with UGDH overexpression was observed, suggesting desmoplasia and fibrosis. Furthermore, the increased expression of inflammatory immune adhesion and signaling mediators Versican, Laminin and IL-6 [70, 71] observed in the ACI23 overexpressing tumors, as well as a trend of increased Nos2 mRNA, and macrophage marker Iba1 expression, suggesting a recruitment of innate immune cells. In vitro, UGDH overexpression in ACI23 cells showed increased expression of cytokines IL-6, IL-8 and CCL2 the latter is also known as monocyte chemoattractant protein-1 (MCP-1), that regulates monocyte and macrophage migration [72]. This suggests suppressed UGDH expression in the C4/DIF subtype limits the activation of a pro-inflammatory stroma that can attract immune cells and become fibrotic. We did not have a syngeneic model of the C1/MES and C4/DIF subtypes to thoroughly examine immune infiltration in xenografts, but our findings warrant further investigation to explore whether UGDH influences immune infiltration in EOC as was recently described in glioblastoma [73].

## Conclusions

UGDH expression in EOC influences the TME and reveals a distinct role for EOC-expressed UGDH in the C1/Mesenchymal and C4/Differentiated molecular subtypes of EOC. UGDH is a strong potential therapeutic target in TICs, for the prevention or treatment of recurrent EOC especially in the mesenchymal subtype.

### Supplementary Information

**Additional file 1: Supplementary Data 1.** Normalized viability data and Z scores of OV90 grown in adherent (2D) or spheroid (3D) conditions for whole-genome siRNA screen used to generate Fig. 1A. Organized by Gene level (average of siRNA values per gene) or by siRNA level (individual siRNAs for genes).

**Additional file 2: Supplementary Table 1.** Correlative analyses by Fisher's exact test of UGDH expression and localization with recurrence and survival status, and progression-free and overall survival analysis by Log-rank(Mantel-Cox) analysis in high grade serous ovarian cancers. Cytoplasmic High=moderate+strong, Low=negative+weak. Nuclear High = high, Low= low + negative.

**Additional file 3: Supplementary Table 2.** Correlative analyses by Fisher's exact test of UGDH expression with FIGO stage, recurrence and survival status, and progression-free and overall survival analysis by Log-rank (Mantel-Cox) analysis in clear cell, endometrioid and mucinous ovarian cancers. High= above the median H score, Low = below the median H-score.

**Additional file 4: Supplementary Table 3.** Analyses of UGDH expression and localization with overall survival (OS) and progression-free survival (PFS) in ovarian cancer molecular subtypes by Log-rank (Mantel-Cox) test.

**Additional file 5: Supplementary Table 4.** Summary of results of functional assays, culture conditions and cell lines used in Figs. 5 and 6. Effects increased or decreased compared to controls.

**Additional file 6: Supplementary Figure 1.** Linear relationship of H-scores for nuclear and cytoplasmic UGDH expression for each tumor tissue sample in molecular subtypes of high grade epithelial ovarian cancers.

**Additional file 7: Supplementary Figure 2. **Effects of UGDH knockdown in OV90 cells, and over-expression in ACI23 cells in vitro. A) Representative brightfield images of adherent cell culture morphology of OV90 control (shneg) and UGDH knockdown (sh459, sh939) cells and B) ACI23 control (VC) and UGDH-overexpressing (OverX) cells. Magnifications as indicated; scale bar is 100µm. C) Representative images of OV90 control cells (shneg) and UGDH silenced cells (sh459, 939) with immunofluorescent staining of E-cadherin (green), Vimentin (purple), and merged images with nuclear stain DAPI (blue) at 60x magnification, scale bar is 20µm. D) Ratio of E-cadherin: Vimentin intensity of immunofluorescent images. *p<0.05, ***p<0.001.

**Additional file 8: Supplementary Figure 3. **Total collagen content of fibroblasts was measured after 3 days culture in conditioned medium from: A) HEYA8 control cells (shneg) or HEYA8 cells with UGDH knocked down (sh459. sh939) compared to culture medium (M), B) SKOV3 cells with UGDH overexpression (OverX) or vector control (VC) compared to culture medium (M). C) Expression of IL-6 and D) IL-8 in supernatant from HEYA8 adherent cells or spheroids with UGDH knockdown (sh459, sh939) compared to controls (shneg). E) Expression of IL-6 and F) IL-8 in supernatant from SKOV3 cells with UGDH overexpression (OverX) or vector control (VC). *p<0.05, **p<0.01, ***p<0.001.

**Additional file 9: Supplementary Figure 4. **Analysis of immune cells and macrophages in ACI23 xenografts UGDH overexpression (OverX) or vector control (VC). A) Number of CD45+ cells per mm^2,^ B) Percentage of CD45+ immune cells. C) Number of Iba1+ cells per mm^2,^ D) Percentage of Iba1+ immune cells. E) Number of iNOS+ cells per mm^2,^ F) Percentage of iNOS+ immune cells. G) Number of CD206+ cells per mm^2,^ H) Percentage of CD206+ immune cells. *p<0.05, **p<0.01, ***p<0.001.

## Data Availability

All data are available in the main text or the supplementary materials.

## Abbreviations

EOC: Epithelial ovarian cancer
UGDH: Uridine diphosphate glucose dehydrogenase
ECM: Extracellular matrix
TICs: Tumor-initiating cells
PARP: Poly ADP-ribose polymerase
DOX: Doxycycline
PBS: Phosphate-buffered saline
IHC: Immunohistochemistry
TME: Tumor microenvironment
GSTA4: Glutathione transferase α4
NAMPT: Nicotinamide phosphoribosyltransferase
NAD: Nicotinamide adenine dinucleotide
GSEA: Gene Set Enrichment Analysis
HGS: High-grade serous
TMA: Tissue microarray
FIGO: International Federation of Gynecology and Obstetrics
C1/MES: C1/Mesenchymal
C2/IMR: C2/Immunoreactive
C4/DIF: C4/Differentiated
C5/PRO: C5/Proliferative
VC: Vector control
OverX: Overexpressed UGDH
ALDH: Aldehyde dehydrogenase
VCAN: Versican
FN1: Fibronectin 1
COL1A1: Collagen, type I, alpha 1
LAMA3: Laminin Subunit Alpha 3
CDH1: E-cadherin
MMP1: Matrix Metallopeptidase 1
TGFB: Transforming Growth Factor-Beta
TIMP3: Tissue Inhibitor of Metalloproteinase 3
Nos2: Nitric oxide synthase-2
IL-6: Interleukin 6
IL8: Interleukin 8
CCL2: C–C Motif Chemokine Ligand 2
