# Supplementary material for: UGDH promotes tumor-initiating cells and a fibroinflammatory tumor microenvironment in ovarian cancer
Source: J Exp Clin Cancer Res. 2023 Oct 19;42:270. doi: 10.1186/s13046-023-02820-z (PMC10585874; doi:10.1186/s13046-023-02820-z)
Supplement: Supplementary file 6 — Additional file 6: Supplementary Figure 1. Linear relationship of H-scores for nuclear and cytoplasmic UGDH expression for each tumor tissue sample in molecular subtypes of high grade epithelial ovarian cancers. [file 13046_2023_2820_MOESM6_ESM.pptx]

## Slide 1
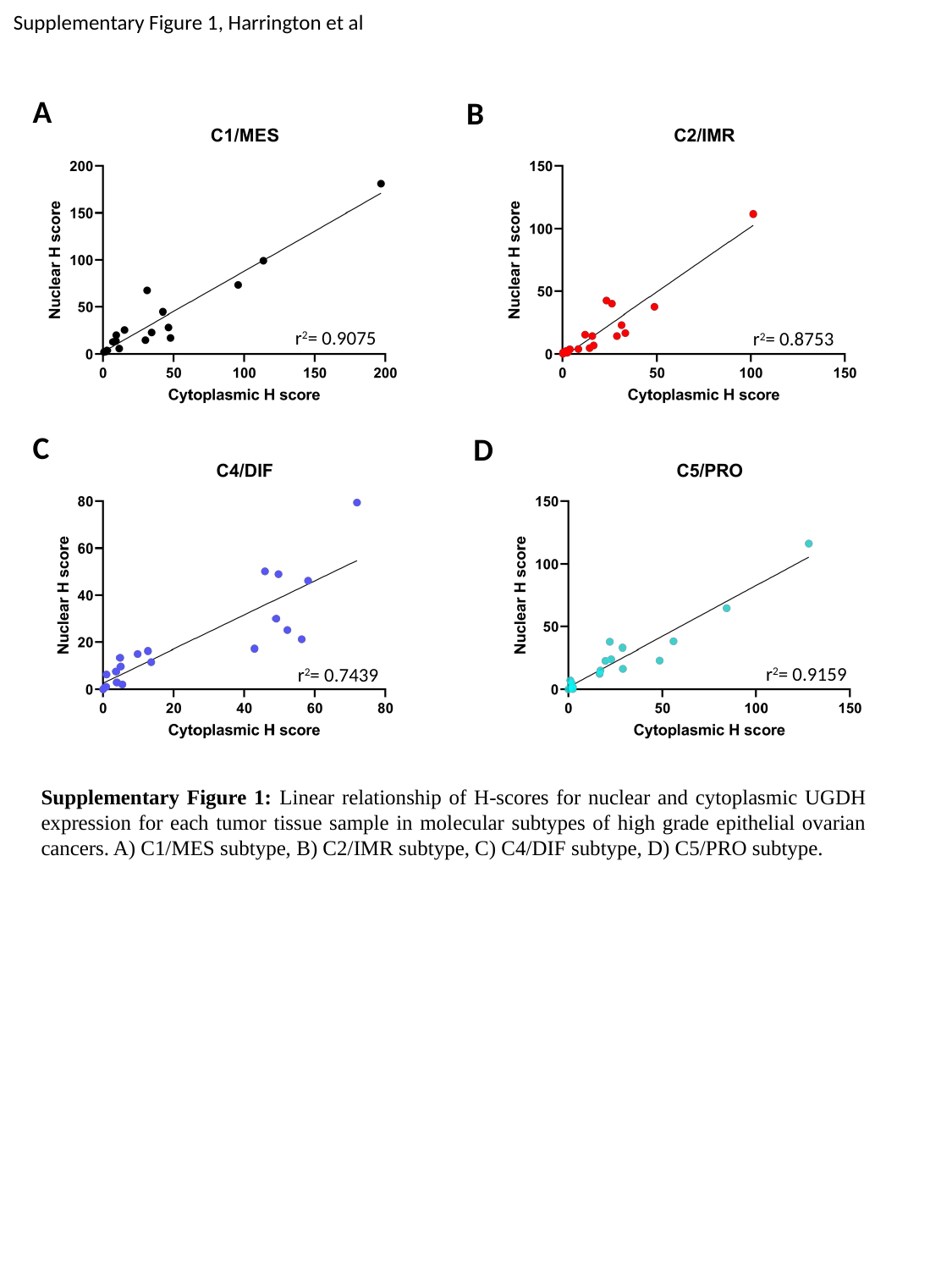

Supplementary Figure 1, Harrington et al
A
B
r2= 0.9075
r2= 0.8753
C
D
r2= 0.7439
r2= 0.9159
Supplementary Figure 1: Linear relationship of H-scores for nuclear and cytoplasmic UGDH expression for each tumor tissue sample in molecular subtypes of high grade epithelial ovarian cancers. A) C1/MES subtype, B) C2/IMR subtype, C) C4/DIF subtype, D) C5/PRO subtype.
