# Supplementary material for: UGDH promotes tumor-initiating cells and a fibroinflammatory tumor microenvironment in ovarian cancer
Source: J Exp Clin Cancer Res. 2023 Oct 19;42:270. doi: 10.1186/s13046-023-02820-z (PMC10585874; doi:10.1186/s13046-023-02820-z)
Supplement: Supplementary file 7 — Additional file 7: Supplementary Figure 2. Effects of UGDH knockdown in OV90 cells, and over-expression in ACI23 cells in vitro. A) Representative brightfield images of adherent cell culture morphology of OV90 control (shneg) and UGDH knockdown (sh459, sh939) cells and B) ACI23 control (VC) and UGDH-overexpressing (OverX) cells. Magnifications as indicated; scale bar is 100µm. C) Representative images of OV90 control cells (shneg) and UGDH silenced cells (sh459, 939) with immunofluorescent staining of E-cadherin (green), Vimentin (purple), and merged images with nuclear stain DAPI (blue) at 60x magnification, scale bar is 20µm. D) Ratio of E-cadherin: Vimentin intensity of immunofluorescent images. *p<0.05, ***p<0.001. [file 13046_2023_2820_MOESM7_ESM.pptx]

## Slide 1
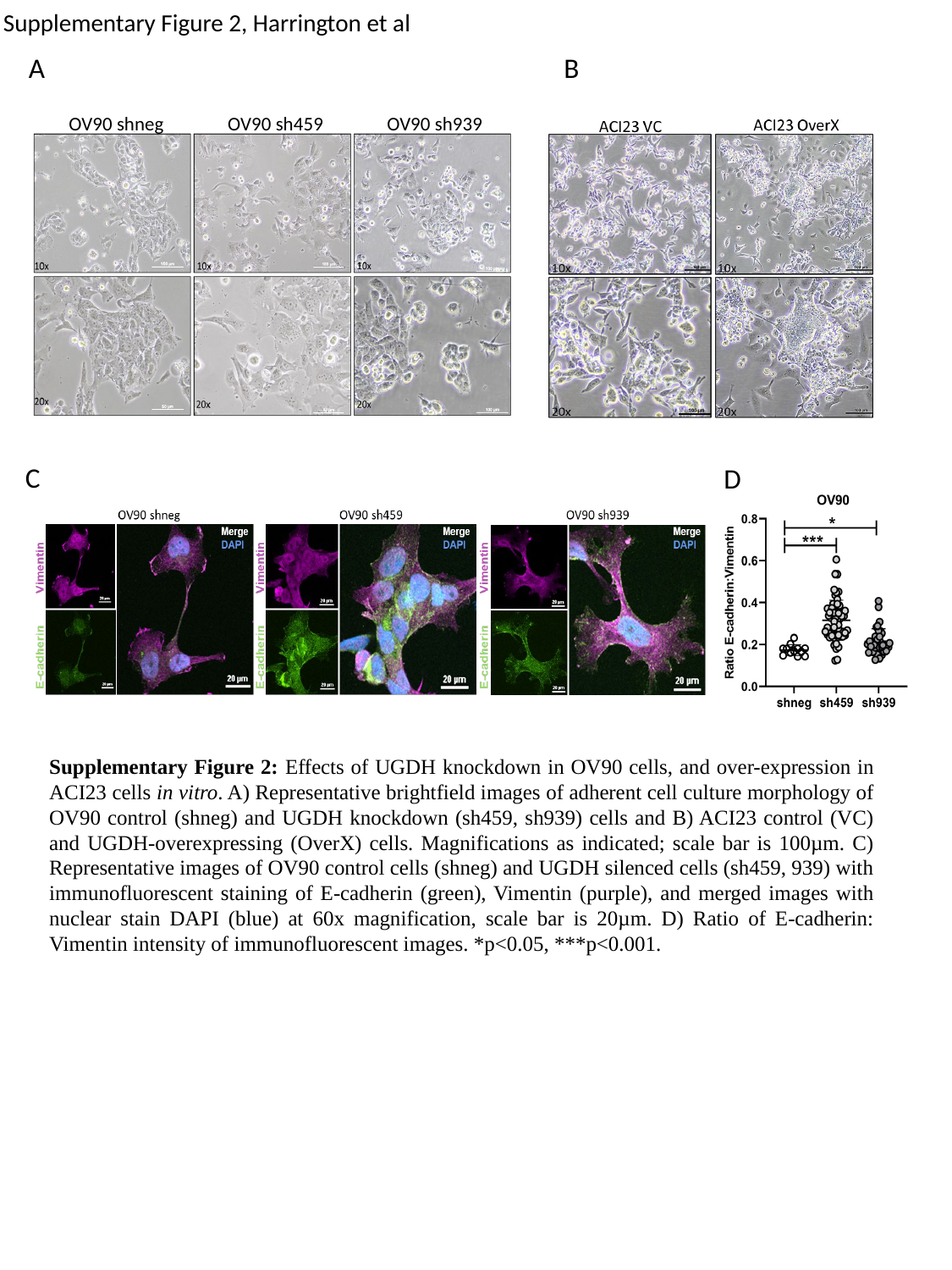

Supplementary Figure 2, Harrington et al
A
B
OV90 shneg
OV90 sh459
OV90 sh939
C
D
Supplementary Figure 2: Effects of UGDH knockdown in OV90 cells, and over-expression in ACI23 cells in vitro. A) Representative brightfield images of adherent cell culture morphology of OV90 control (shneg) and UGDH knockdown (sh459, sh939) cells and B) ACI23 control (VC) and UGDH-overexpressing (OverX) cells. Magnifications as indicated; scale bar is 100µm. C) Representative images of OV90 control cells (shneg) and UGDH silenced cells (sh459, 939) with immunofluorescent staining of E-cadherin (green), Vimentin (purple), and merged images with nuclear stain DAPI (blue) at 60x magnification, scale bar is 20µm. D) Ratio of E-cadherin: Vimentin intensity of immunofluorescent images. *p<0.05, ***p<0.001.
