# Supplementary material for: UGDH promotes tumor-initiating cells and a fibroinflammatory tumor microenvironment in ovarian cancer
Source: J Exp Clin Cancer Res. 2023 Oct 19;42:270. doi: 10.1186/s13046-023-02820-z (PMC10585874; doi:10.1186/s13046-023-02820-z)
Supplement: Supplementary file 8 — Additional file 8: Supplementary Figure 3. Total collagen content of fibroblasts was measured after 3 days culture in conditioned medium from: A) HEYA8 control cells (shneg) or HEYA8 cells with UGDH knocked down (sh459. sh939) compared to culture medium (M), B) SKOV3 cells with UGDH overexpression (OverX) or vector control (VC) compared to culture medium (M). C) Expression of IL-6 and D) IL-8 in supernatant from HEYA8 adherent cells or spheroids with UGDH knockdown (sh459, sh939) compared to controls (shneg). E) Expression of IL-6 and F) IL-8 in supernatant from SKOV3 cells with UGDH overexpression (OverX) or vector control (VC). *p<0.05, **p<0.01, ***p<0.001. [file 13046_2023_2820_MOESM8_ESM.pptx]

## Slide 1
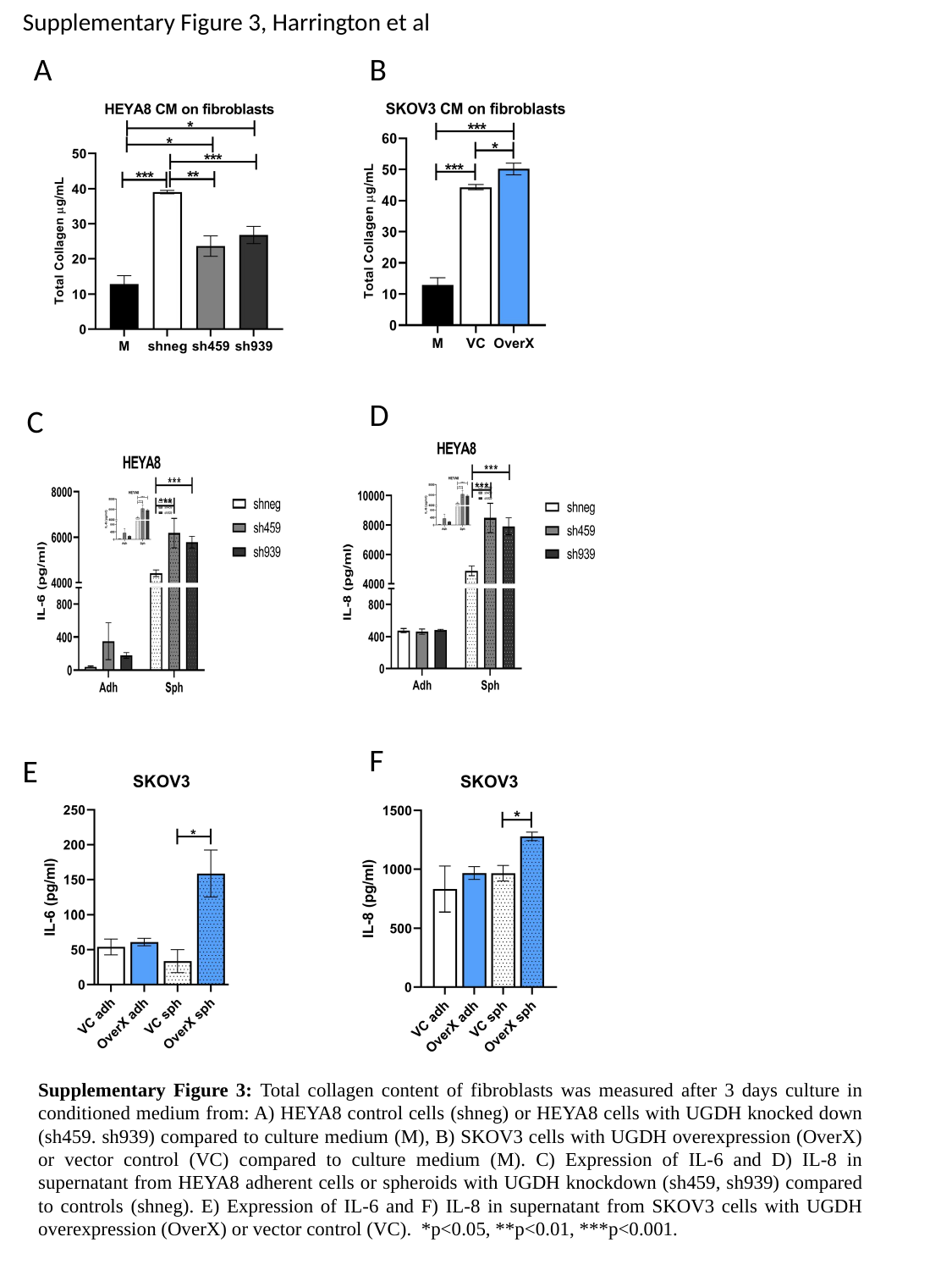

Supplementary Figure 3, Harrington et al
A
B
D
C
F
E
Supplementary Figure 3: Total collagen content of fibroblasts was measured after 3 days culture in conditioned medium from: A) HEYA8 control cells (shneg) or HEYA8 cells with UGDH knocked down (sh459. sh939) compared to culture medium (M), B) SKOV3 cells with UGDH overexpression (OverX) or vector control (VC) compared to culture medium (M). C) Expression of IL-6 and D) IL-8 in supernatant from HEYA8 adherent cells or spheroids with UGDH knockdown (sh459, sh939) compared to controls (shneg). E) Expression of IL-6 and F) IL-8 in supernatant from SKOV3 cells with UGDH overexpression (OverX) or vector control (VC). *p<0.05, **p<0.01, ***p<0.001.
