# Supplementary material for: UGDH promotes tumor-initiating cells and a fibroinflammatory tumor microenvironment in ovarian cancer
Source: J Exp Clin Cancer Res. 2023 Oct 19;42:270. doi: 10.1186/s13046-023-02820-z (PMC10585874; doi:10.1186/s13046-023-02820-z)
Supplement: Supplementary file 9 — Additional file 9: Supplementary Figure 4. Analysis of immune cells and macrophages in ACI23 xenografts UGDH overexpression (OverX) or vector control (VC). A) Number of CD45+ cells per mm2, B) Percentage of CD45+ immune cells. C) Number of Iba1+ cells per mm2, D) Percentage of Iba1+ immune cells. E) Number of iNOS+ cells per mm2, F) Percentage of iNOS+ immune cells. G) Number of CD206+ cells per mm2, H) Percentage of CD206+ immune cells. *p<0.05, **p<0.01, ***p<0.001. [file 13046_2023_2820_MOESM9_ESM.pptx]

## Slide 1
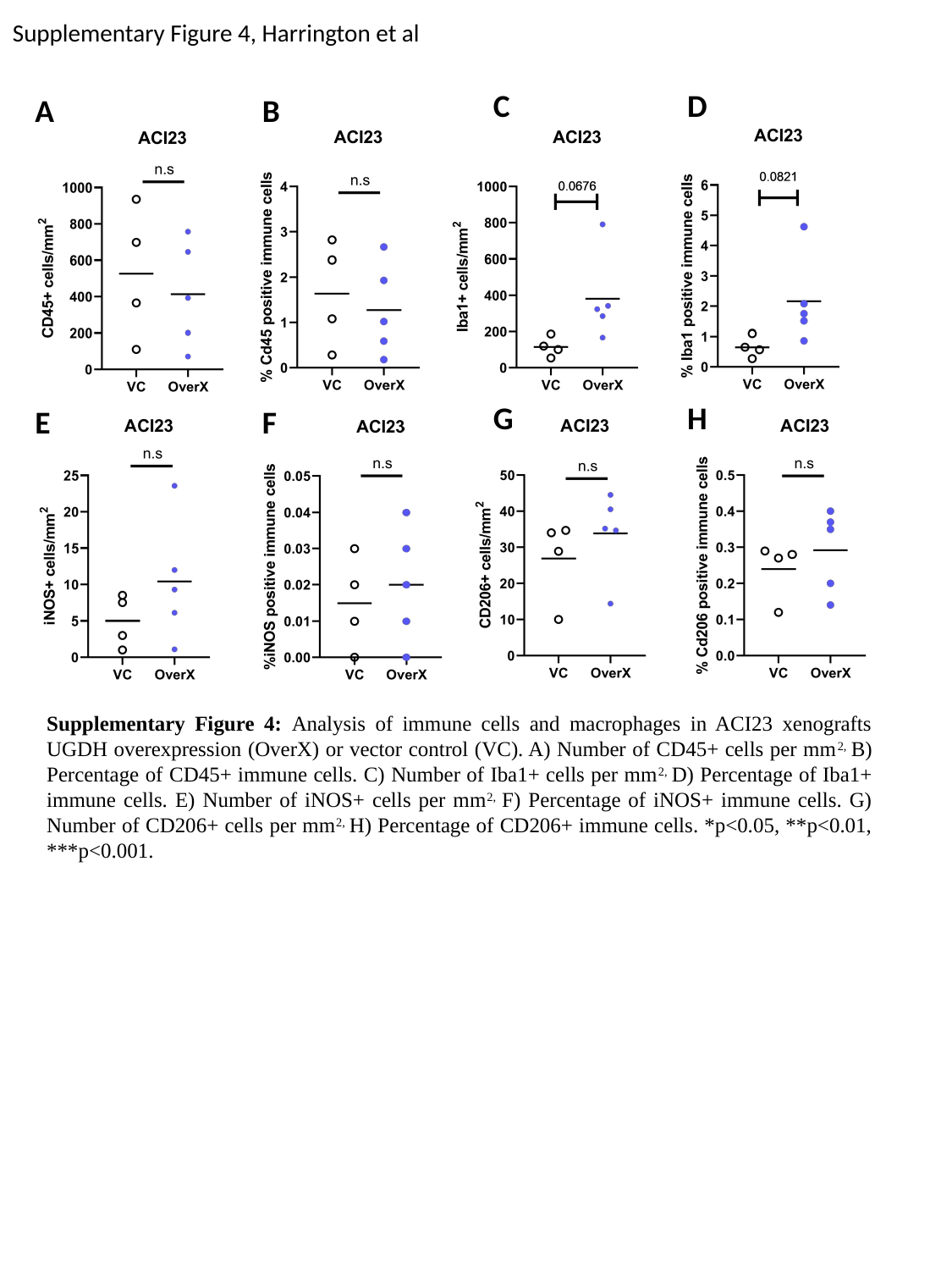

Supplementary Figure 4, Harrington et al
C
D
A
B
G
H
E
F
Supplementary Figure 4: Analysis of immune cells and macrophages in ACI23 xenografts UGDH overexpression (OverX) or vector control (VC). A) Number of CD45+ cells per mm2, B) Percentage of CD45+ immune cells. C) Number of Iba1+ cells per mm2, D) Percentage of Iba1+ immune cells. E) Number of iNOS+ cells per mm2, F) Percentage of iNOS+ immune cells. G) Number of CD206+ cells per mm2, H) Percentage of CD206+ immune cells. *p<0.05, **p<0.01, ***p<0.001.
